# An EEG-based attention recognition method: fusion of time domain, frequency domain, and non-linear dynamics features

**DOI:** 10.3389/fnins.2023.1194554

**Published:** 2023-07-12

**Authors:** Di Chen, Haiyun Huang, Xiaoyu Bao, Jiahui Pan, Yuanqing Li

**Affiliations:** ^1^School of Automation Science and Engineering, South China University of Technology, Guangzhou, China; ^2^Research Center for Brain-Computer Interface, Pazhou Laboratory, Guangzhou, China; ^3^School of Software, South China Normal University, Foshan, China

**Keywords:** electroencephalogram (EEG), brain-computer interfaces (BCIs), attention recognition, valid paradigm, intra-subject, inter-subject, neural patterns

## Abstract

**Introduction:**

Attention is a complex cognitive function of human brain that plays a vital role in our daily lives. Electroencephalogram (EEG) is used to measure and analyze attention due to its high temporal resolution. Although several attention recognition brain-computer interfaces (BCIs) have been proposed, there is a scarcity of studies with a sufficient number of subjects, valid paradigms, and reliable recognition analysis across subjects.

**Methods:**

In this study, we proposed a novel attention paradigm and feature fusion method to extract features, which fused time domain features, frequency domain features and nonlinear dynamics features. We then constructed an attention recognition framework for 85 subjects.

**Results and discussion:**

We achieved an intra-subject average classification accuracy of 85.05% ± 6.87% and an inter-subject average classification accuracy of 81.60% ± 9.93%, respectively. We further explored the neural patterns in attention recognition, where attention states showed less activation than non-attention states in the prefrontal and occipital areas in α, β and θ bands. The research explores, for the first time, the fusion of time domain features, frequency domain features and nonlinear dynamics features for attention recognition, providing a new understanding of attention recognition.

## 1. Introduction

Attention is a crucial cognitive process that allows individuals to selectively focus on specific aspects of their environment while filtering out irrelevant information, thereby enabling effective adaptation to their surroundings (Petersen and Posner, 2012). Poor attention and concentration skills can contribute to mental health problems such as anxiety and depression. If left unaddressed, these difficulties can develop into more severe conditions such as attention deficit hyperactivity disorder (ADHD) (Chen et al., 2019). Attention recognition is an emerging research area that provides a window to monitor and understand people's attention states. It shows significant potential application value in the fields of medicine (Moghaddari et al., 2020), military operations (Berka et al., 2004), and preventing fatigue while driving (Luo et al., 2019). Existing research methods for attention recognition have focused on psychological behavior scale tests, such as digital cancelation task (D-CAT) (Fliege et al., 2009) and simple reaction time (SRT) Krupski and Boyle (1978), Combined Raven's Test (CRT) (Wang et al., 1989), Shure grid test scale, and Conners et al. (1998). However, these methods are limited in their ability to provide real-time results of a user's attention states. Therefore, researchers turned to explore attention recognition based on neurophysiological signals with temporal information, such as heart rate, skin electricity, electroencephalogram (EEG), functional magnetic resonance imaging (fMRI), or multimodal methods, which captured physiological changes related to attention states. Compared to other peripheral physiological signals, EEG signal, with their high temporal resolution, provided more information about attention and showed great potential in the field of attention recognition (Andrillon et al., 2021).

Research on attention recognition based on EEG signal is of significant practical importance in brain–computer interface (BCI) applications. In a study by Hamadicharef Hamadicharef et al. (2009), they used a combination of temporal filters, spatial filters, and Fisher linear discriminant to classify attention states within five subjects and achieved an accuracy of 89.4%. Mohammadpour built an EEG-based BCI, which successfully recognized four levels of attention in five individuals with an accuracy of 63.5% (Mohammadpour and Mozaffari, 2017). Ac achieved an accuracy of 91.72% within subjects using time-frequency features and SVM in 2019 (Acı et al., 2019), while in 2021, Wang wan obtained an accuracy of 95.36% ± 2.31% for two attention levels within subjects using dynamical complexity (Wan et al., 2021). In light of the preceding information, studies in EEG-based attention recognition typically have fewer subjects than studies in other EEG fields (Zheng et al., 2017; Gao et al., 2021), which results in insufficient generalizablation on unseen data and obstacles in inter-subject studies.

Attention recognition paradigms based on EEG commonly involve the use of cues to prompt subjects to enter a state of attention or relaxation. The state of attention is typically associated with a task state, while the state of relaxation is considered a task-independent state. Tasks used to induce a state of attention include breath counting Braboszcz and Delorme (2011); Hosseini and Guo (2019), reading comprehension (Li et al., 2011), mental arithmetic (Hamadicharef et al., 2009), imagination (Ke et al., 2014), and Stroop test (Kawashima et al., 2023). However, how to induce participants under specific cognitive load and enhance their attention is still a challenging work.

Brain waves can be divided into different frequency bands, including δ (0.5–4Hz), θ (4–8Hz), α (8–13Hz), β (13–30Hz), and γ (30–50Hz), each of which is associated with specific physiological functions. Previous research showed that these frequency bands can reflect attention needs, emotional states, and cognitive processes (Rao, 2013). For example, studies by Ray demonstrated that EEG activities are related to attention (Ray and Cole, 1985). Klimesch and other researchers found that α wave amplitudes are smaller when individuals focused on mental arithmetic tasks Klimesch et al. (1993). Despite these findings, the specific neural patterns underlying attention-related EEG activities still require further investigation.

The application of EEG-based BCI in attention recognition is currently in its nascent stages. Researchers attempted to employ feature extraction methods such as power spectrum or non-linear dynamics (including approximate entropy and sample entropy) to identify attention levels. However, the application of attention recognition across subjects is hindered due to the lack of significant datasets, effective paradigms, and comprehensive feature analysis.

In this study, we first proposed a novel attention paradigm based on mental arithmetic tasks and built an EEG dataset of 85 subjects for attention recognition. Second, we proposed a composite EEG-based feature that took time domain, frequency domain, and non-linear dynamic features into consideration and constructed an attention recognition framework both across and within subjects. The best intra-subject accuracy and inter-subject accuracy are 85.05% ± 6.87% and 81.60% ± 9.93%, respectively. Furthermore, we explored neural patterns within attention and non-attention states and found that attention states showed less activation than non-attention states in the prefrontal and occipital areas across α, β, and θ bands.

This study is organized into five sections. Materials and Methods are presented in Section 2. Experimental results are presented in Section 3. Discussion is available in Section 4. Conclusion is presented in Section 5.

## 2. Materials and methods

**Figure 1:**
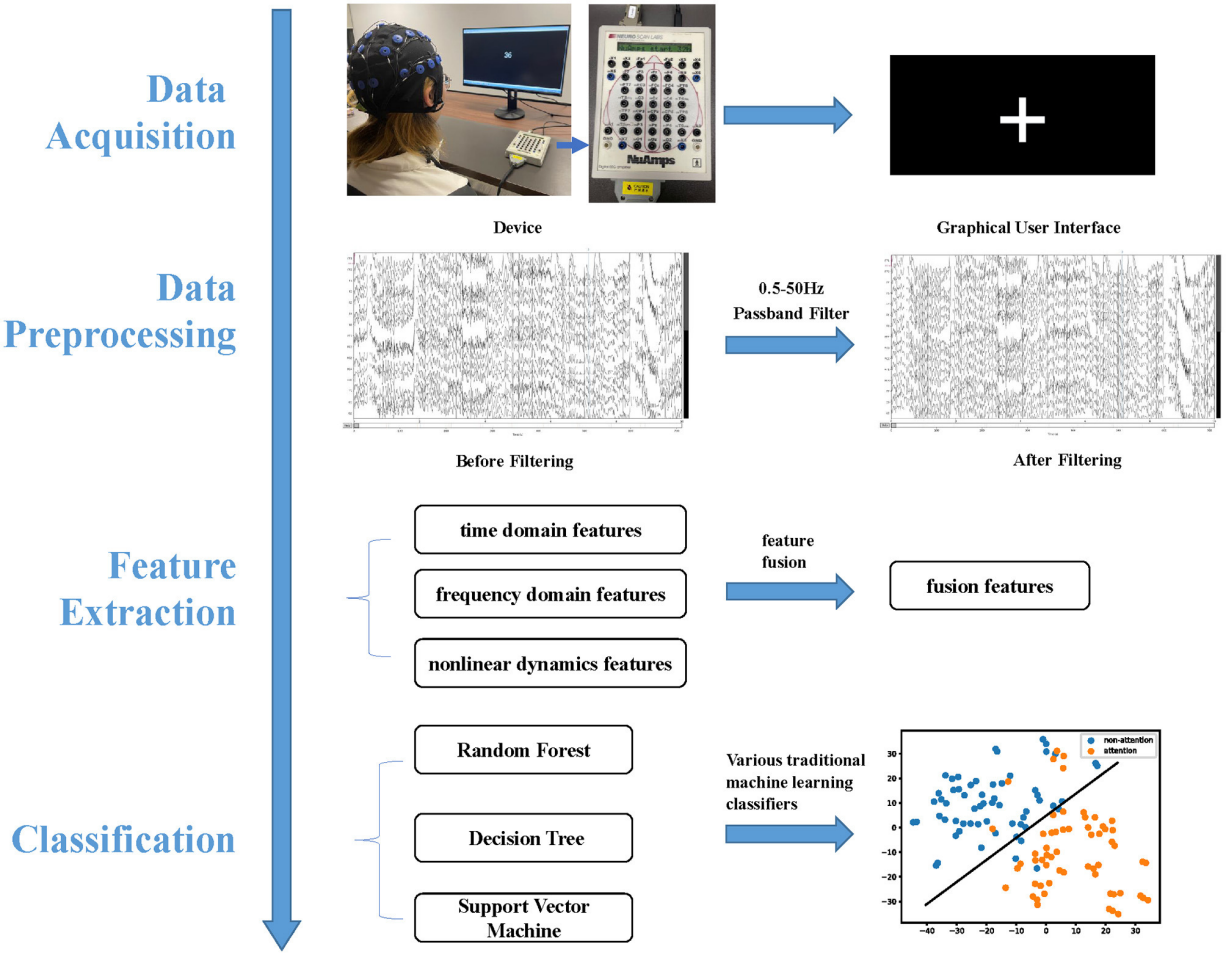
Attention recognition framework is comprised of four integral components, namely data acquisition, data preprocessing, feature extraction, and classification.

In this section, we collected EEG data, conducted a preprocessing of raw EEG data, fused three types of features, and classified features for both intra- and inter-subject attention recognition. Additionally, we analyzed common neural patterns between attention and non-attention across subjects. Figure 1 depicts the attention recognition analysis framework, which encompasses EEG acquisition, data preprocessing, feature extraction, and classification.

### 2.1. Data acquisition

#### 2.1.1. Equipment

**Figure 2:**
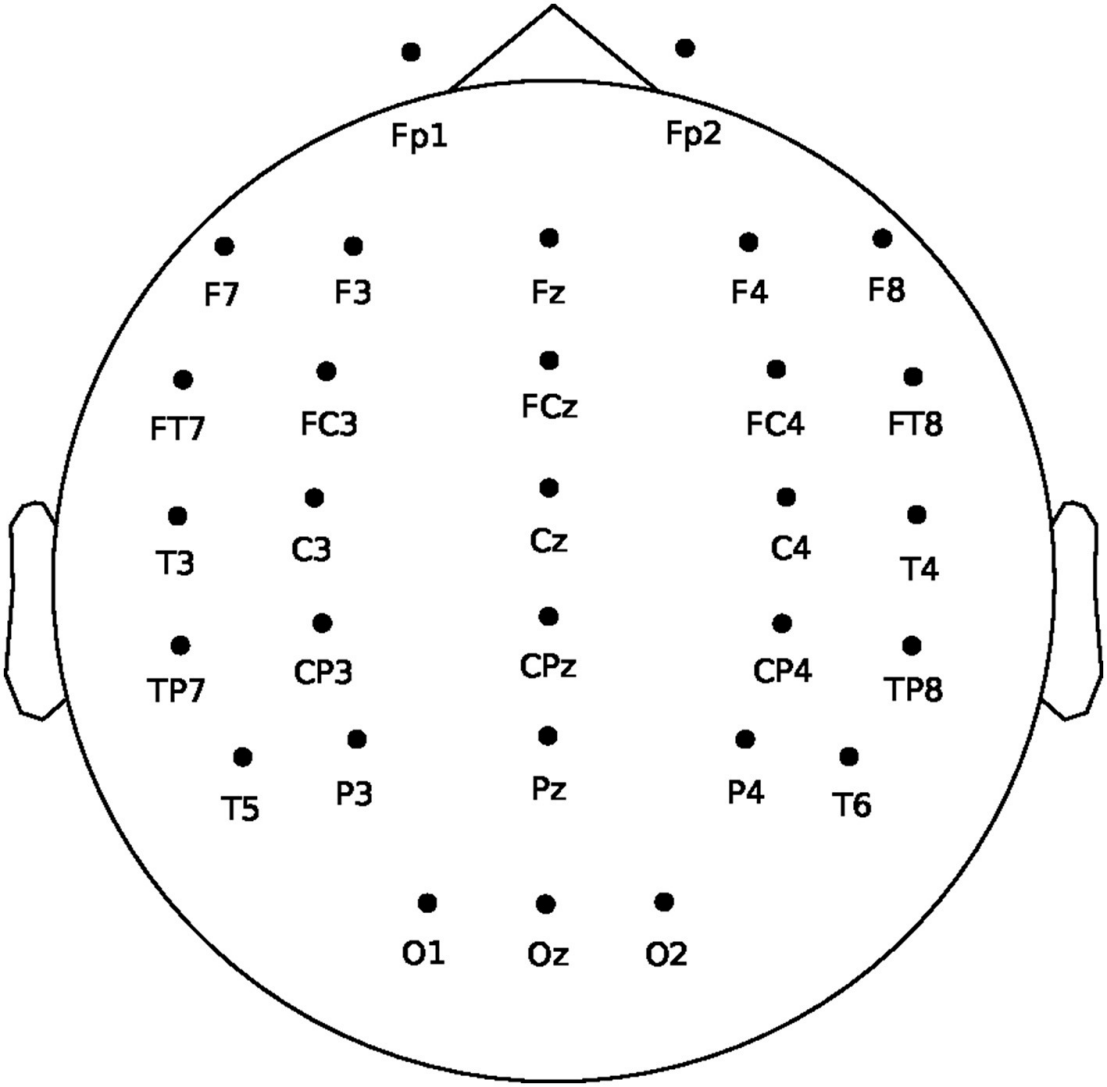
EEG cap layout for 32 channels.

Our study collected EEG signals using a 32-channel Neuroscan amplifier in accordance with the international 10–20 system (Gao et al., 2021). The signals were sampled at a rate of 250Hz and band-pass filtered between 0.1 and 50 Hz. Figure 2 displays the layout of EEG electrodes on the cap. To obtain high-quality data, we ensured that the impedance of each electrode was below 5 kΩ. The experiment employed a 22-inch external screen as a monitor and utilized the computer with a 32-bit Windows 7 system to store EEG data and run interface programs.

#### 2.1.2. Subjects

Eighty-five subjects with healthy visual and cognitive abilities were recruited from various universities in Guangzhou for this experiment. The subjects had an average age of 25.3 ± 2.4, with 45 male subjects and 40 female subjects. According to the Edinburgh Handedness Inventory (Robinson, 2021), all of these subjects were right-handed, and none had prior experience with attention-related BCI experiments. Moreover, all subjects were informed about the content and purpose of the study, and informed consent was obtained.

#### 2.1.3. Paradigm

In the absence of a standard experimental paradigm for attention recognition, we proposed our own based on mental arithmetic and resting tasks for our study. Mental arithmetic tasks in our experiment were able to induce cognitive load on participants and improved their attention (Chin et al., 2018). Mental arithmetic tasks require cognitive resources such as working memory, attention control, and executive function (Hester and Garavan, 2005). When performing mental arithmetic, individuals need to retrieve numerical information from long-term memory, hold that information in working memory, manipulate that information to perform calculations, and monitor their progress toward a solution (Grabner and De Smedt, 2011).

**Figure 3:**
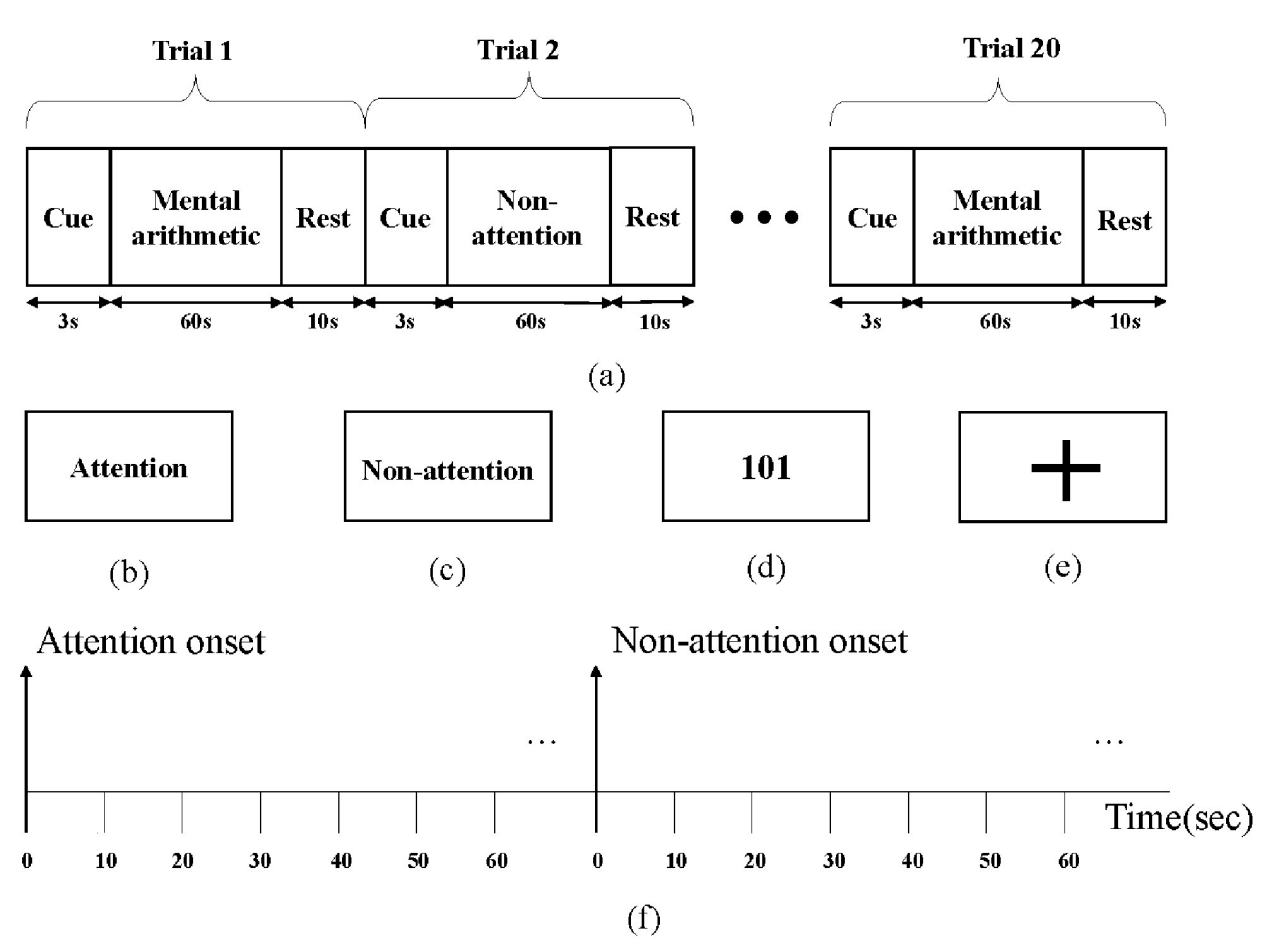
Attention paradigm: **(a)** the recording protocol, **(b)** attention cue in screen, **(c)** non-attention cue in screen, **(d)** mental arithmetic task, **(e)** non-attention task, and **(f)** a segmentation diagram.

The subjects were seated in a quiet room, and their brain activity was measured using an EEG acquisition device during the experiment. Figure 3 depicts the experimental paradigm, which contains 20 trials. Each trial includes a 3s cue, a 60s task, and a 10s rest period. Subjects were asked to prepare to enter the attention or non-attention state based on the screen cue during the cue period. The attention cue is depicted in Figure 3b, while the non-attention cue is depicted in Figure 3c. During the attention target state, subjects were instructed to keep doing mental arithmetic, which followed the rhythm of the screen, while a random number (possibly positive or negative) appeared on the screen and were continuously subtracted by 3 over time, as shown in Figure 3d. During the non-attention state, a fixed plus sign appeared on the screen as shown in Figure 3e; meanwhile, the subjects were asked to rest quietly with their eyes open. At the end of each trial, the subjects would be given 10 seconds to rest, called rest period. Each experimental session contained 10 attention states and 10 non-attention states, with the order randomized.

### 2.2. Data preprocessing

The raw EEG signals were first filtered by a finite impulse response (FIR) band-pass filter between 0.5 Hz and 50 Hz to reduce noise and extract relevant information. Next, 15,000-point data (60s) for each trial in each channel were equally cut into 6 epochs of equal length, each containing 2,500-point data (10 s), as illustrated in Figure 3f. By the above operations, the data of each subject containing 20 trials were transformed into 120 epochs, of which 60 epochs corresponded to the labels of attention states and the other 60 epochs corresponded to the labels of non-attention states. To ensure the quality of the data, epochs with high amplitude or significant myoelectricity were removed from the dataset. After preprocessing, a total of 10,188 epochs were obtained from 85 subjects.

### 2.3. Feature extraction

It is now generally accepted that advanced cognition in the brain is often associated with time–frequency and non-linear dynamic features of EEG (Klimesch et al., 1993, 1998; Chun et al., 2011). To comprehensively investigate the relationship between these features and attention states, we exhaustively enumerated and extracted time domain features, frequency domain features, and non-linear dynamic features from the preprocessed EEG data, as described in Table 1. To simplify notation, we utilized *T*_*l*_, *F*_*l*_, and *D*_*l*_ to represent a time domain feature, a frequency domain feature, and a non-linear dynamics feature, respectively, with the subscript *l* denoting the order of the feature in Table 1. Additionally, we used $T_{l}^{m}$, $F_{l}^{m}$, and $D_{l}^{m}$ to signify the *m*-th dimension of the time domain feature *T*_*l*_, frequency doamin feature *F*_*l*_, and non-linear dynamic features *D*_*l*_, respectively. For example, $F_{4}^{3}$ denotes the third dimension of power spectrum for frequency bands *F*_4_.

**Table 1:** List of representative EEG features extracted in our works.

**Feature Type**	**Extracted Features**	**Feature Size**	**References**
	1. Standard deviation.	1	Hjorth, 1970
	2. Mean.	1	Dumermuth and Molinari, 1987
	3. Peak-to-Peak Amplitude.	1	Barry et al., 2000
	4. Skewness.	1	Pollock et al., 1990
	5. Kurtosis.	1	Delorme et al., 2001
time domain features	6. Root-Mean Squared Value.	1	Lykken et al., 1974
	7. Hjorth Parameter: Mobility.	1	Päivinen et al., 2005
	8. Quantile	1	Grieszbach and Schack, 1993
	9. Hjorth Parameter: Complexity.	1	Päivinen et al., 2005
	10. Variance.	1	Dumermuth and Molinari, 1987
	11. Decorrelation Time.	1	Teixeira et al., 2011
	12. Number of zero-crossings.	1	Borbely and Neuhaus, 1979
	1. Harmonic Parameters.	5	Van Hese et al., 2001
	2. Energy of Wavelet decomposition coefficients.	6	Teixeira et al., 2011
	3. Hjorth complexity parameter by the Power Spectrum.	1	Mormann et al., 2007
	4. Power Spectrum for frequency bands.	15	Teixeira et al., 2011
frequency domain features	5. Linear regression of the the log–log frequency curve.	4	Demanuele et al., 2007
	6. Hjorth mobility parameter by the Power Spectrum.	1	Mormann et al., 2007
	7. Spectal Edge Frequency.	1	Schwender et al., 1996
	8. Band Energy.	5	Kharbouch et al., 2011
	9. Median Frequency.	10	Gudmundsson et al., 2005
	1. Petrosian Fractal Dimension.	1	Mardi et al., 2011
	2. Line length.	1	Esteller et al., 2001
	3. Spectral Entropy.	1	Inouye et al., 1991
	4. Hurst Exponent.	1	Kannathal et al., 2005
	5. Sample Entropy.	1	Bai et al., 2007
	6. Renyi Entropy.	10	Tong et al., 2003
	7. Tsallis Entropy.	10	Capurro et al., 1998
	8. Shannon entropy.	10	Papadelis et al., 2006
non-linear dynamic features	9. Approximate Entropy.	1	Srinivasan et al., 2007
	10. SVD entropy.	1	Roberts et al., 1999
	11. Permutation Entropy.	1	Li et al., 2010
	12. Higuchi Fractal Dimension.	1	Spasic et al., 2011
	13. Wavelet Entropy.	7	Rosso et al., 2001
	14. Teager–kaiser energy.	14	Badani et al., 2017
	15. SVD Fisher Information.	1	Roberts et al., 1999
	16. Detrended fluctuation analysis.	1	Márton et al., 2014
	17. Katz Fractal Dimension.	1	Esteller et al., 1999

#### 2.3.1. Time domain features

As EEG signals are time-series signals, time domain features (such as mean *T*_2_, skewness *T*_4_, root-mean squared value *T*_6_, standard deviation *T*_10_, and number of zero-crossings *T*_12_) have great advantages in expressing the amplitude, time scale, and complexity of signals. Numerous studies have shown that these features can distinguish different mental states (Vourkas et al., 2000; Wang and Guan, 2008). Furthermore, time domain features have the added advantage of low computational complexity and real-time calculation capability (Hu et al., 2016). Therefore, it is worth exploring time domain features for attention recognition based on EEG. Specifically, some time domain features used in this study are defined as follows.

The formulas of Hjorth parameter (activity (*h*_1_), mobility (*h*_2_), and complexity (*h*_3_)) are defined as follows:


(1) $$\begin{array}{l} h_{1}=\sigma_{x}^{2}, \end{array}$$

(2) $$\begin{array}{l} h_{2}=\sigma_{d}/\sigma_{x}, \end{array}$$

(3) $$\begin{array}{l} h_{\text{3}}=\frac{\sigma_{dd}}{\sigma_{d}}/\frac{\sigma_{d}}{\sigma_{x}}=\sigma_{dd}/\sigma_{x}, \end{array}$$

where $\sigma_{x}^{2}$ is the variance of the signal, σ_*d*_ is the standard deviation of the first derivative of the signal, and σ_*dd*_ is the standard deviation of the second derivative of the signal.

#### 2.3.2. Frequency domain features

Frequency domain features are commonly used in EEG research and have shown great potential in advanced cognitive recognition, such as emotion recognition (Huang W. et al., 2021). In this study, we selected the common EEG rhythms of δ, θ, α, β, and γ as the target frequency bands for analysis. Several frequency domain features were employed in this study, and their partial definitions are presented below.

The power spectrum density (PSD) of these bands *F*_4_, along with their respective ratios, was utilized as features in the upcoming study. Assuming that *X*_*k*_ represents the Fourier transform of the time series *x*[*n*], the relevant PSD *P* is defined as follows:


(4) $$\begin{array}{l} P=\overset{N-1}{\underset{n=0}{\sum }}|x^{2}|=\frac{1}{N}\overset{N-1}{\underset{k=0}{\sum }}|X_{k}^{2}|. \end{array}$$

Median frequency *F*_9_ represents the frequency point that divides the power spectrum band of a signal into two equal parts (Thongpanja et al., 2013). This can be expressed by the following equation:


(5) $$\begin{array}{l} \overset{\text{MF}}{\underset{j=1}{\sum }}P_{j}=\overset{M}{\underset{j=\text{MF}}{\sum }}P_{j}=\frac{1}{2}\overset{M}{\underset{j=1}{\sum }}P_{j}, \end{array}$$

where *P*_*j*_ denotes the power spectrum at frequency bin *j*, and *MF* denotes median frequency. The frequency band is from 1 to M, where 1 < *MF*<*M*. According to a previous study on the application of median frequency to EEG (Gudmundsson et al., 2005), median frequency of 10 frequency bands (0.5–2 Hz, 2–4 Hz, 4–5 Hz, 5–7 Hz, 7–10 Hz, 10–13 Hz, 13–15 Hz, 15–20 Hz, 20–30 Hz, and 30–40 Hz) were calculated in our study.

Discrete wavelet transform can be defined as follows (Blanco et al., 1998):


(6) $$\begin{array}{l} C\left(j,k\right)=\overset{\infty }{\underset{-\infty }{\int }}x\left(t\right)\frac{1}{\sqrt{2^{j}}}\psi \left(\frac{t-2^{j}k}{2^{j}}\right)dt, \end{array}$$

where 2^*j*^*k* and 2^*j*^ represent the time positioning and scale coefficients respectively, and ψ(*t*) represents the mother wavelet function. The energy of each resolution level *j* = 1, ⋯ , *J* by wavelet coefficients can be (Candra et al., 2015) defined as follows:


(7) $$\begin{array}{l} E_{j}=\overset{N}{\underset{k=1}{\sum }}|C_{j,k}{|}^{2},k=1,\ldots ,N, \end{array}$$

where *N* is the number of wavelet coefficients in each decomposition layer. *E*_*j*_ can also be called Wavelet Coef Energy *F*_9_. In this study, we used the mother wavelet Daubechies with a decomposition level of 6, which means *J* = 6.

#### 2.3.3. Non-linear dynamic features

The theory of non-linear dynamics opened up a new window for understanding EEG. One of the non-linear estimates of dynamic EEG activity is complexity analysis. Among all complexity analysis methods, entropy proved to be a useful and robust estimation method for evaluating the regularity or predictability of EEG. The 17 features presented in Table 1 for each channel were calculated in preparation for the next step. Here are some definitions for non-linear dynamic features.

Teager–Kaiser energy *D*_14_ is a non-linear energy tracking method can calculate the instantaneous energy of non-stationary signals (Solnik et al., 2010). For the case of the discrete signals *x*[*n*], the Teager–Kaiser energy ψ can be expressed as (O'Toole et al., 2014; Badani et al., 2017):


(8) $$\begin{array}{l} \psi \left(x\left[n\right]\right)=x^{2}\left[n\right]-x\left[n-1\right]x\left[n+1\right]. \end{array}$$

We used the mean and standard deviation of Teager–Kaiser energy as features for the wavelet transform coefficients of decomposition level 6, which contained seven sets coefficients. This resulted in 14 (2 × 7) dimensional features.

To normalize the energy *E*_*j*_ of resolution level *j*, as calculated from Equation 7, the energy of the fixed resolution level *j* is compared with the total energy of the signal *E*_*t*_:


(9) $$\begin{array}{l} p_{j}=\frac{E_{j}}{E_{t}}, \end{array}$$

where *E*_*t*_ represents the sum of all frequency bands energy, *E*_*j*_ represents the energy of the fixed resolution level *j*, and *p*_*j*_ represents the proportion of *E*_*j*_ to *E*_*t*_. According to Rosso et al. (2001), wavelet entropy (*D*_13_) *H*_*j*_ can be defined as follows:


(10) $$\begin{array}{l} H_{j}=-\sum p_{j}\ln\;p_{j}. \end{array}$$

#### 2.3.4. Feature fusion

Feature-level fusion involves integrating low-level or intermediate-level features extracted from different sources or modalities into a single representation before further analysis or decision-making (Cai et al., 2020). It aims to capture comprehensive and discriminative information provided by multiple features to enhance the overall representation and improve subsequent processing tasks (Chin et al., 2014).

We directly concatenated and fused the features extracted from time domain, frequency domain, and non-linear dynamics analysis methods. Considering that time domain features, frequency-domain features, and non-linear features can be represented as $T\in {\mathbb{R}}^{T_{d}}$, $F\in {\mathbb{R}}^{F_{d}}$, and $D\in {\mathbb{R}}^{D_{d}}$ respectively, the fusion features after concatenation can be represented as follows:


(11) $$\begin{array}{l} Feature_{fusion}=\left[T_{1},T_{2},\ldots ,T_{T_{d}},F_{1},F_{2},\ldots ,F_{F_{d}},D_{1},D_{2},\ldots ,D_{D_{d}}\right], \end{array}$$

where *T*_*d*_, *F*_*d*_, and *D*_*d*_ represent the dimension of time domain features, frequency domain features, and non-linear dynamic features, respectively.

After extracting 12-dimensional time domain features, 48-dimensional frequency domain features, and 63-dimensional non-linear dynamic features, we concatenated them to form fusion features, resulting in a total of 123 dimensions.

### 2.4. Classification

To valid the effectiveness of these extracted features, we used three common classification methods, including random forest (RF), decision tree (DT), and support vector machine (SVM) to build our attention recognition framework.

RF classifiers have been shown to be highly effective in small EEG data sets as demonstrated by the studies conducted by Amin et al. (2017), Lotte et al. (2018). In this study, the random forest classifier held 100 evaluators and used Gini impurity to measure the quality of a split as criterion. For DT, the criterion and splitter were set to information entropy and best. Linear kernel with a penalty parameter C of 2 and the kernel function coefficients of 0.2 was used for constructing the SVM classifiers.

We employed 5-fold cross-validation (CV) for intra-subject classification, dividing the training set and testing set strictly in the order of time. For inter-subject classification, leave-one-subject-out (LOSO) CV was employed.

## 3. Results

### 3.1. Performance of intra-subject attention recognition

In this section, we compared four types of features, namely time domain features, frequency domain features, non-linear dynamic features, and fusion features, in three different classifiers: RF, DT, and SVM. It should be noted that fusion features involve time domain, frequency domain, and non-linear dynamic features.

For each type of feature and each classifier, we calculated the mean accuracy based on a 5-fold CV approach, resulting in 85 mean accuracies in total. We then performed paired-sample *t*-tests to compare the accuracies of fusion features with those of the other types of features.

**Figure 4:**
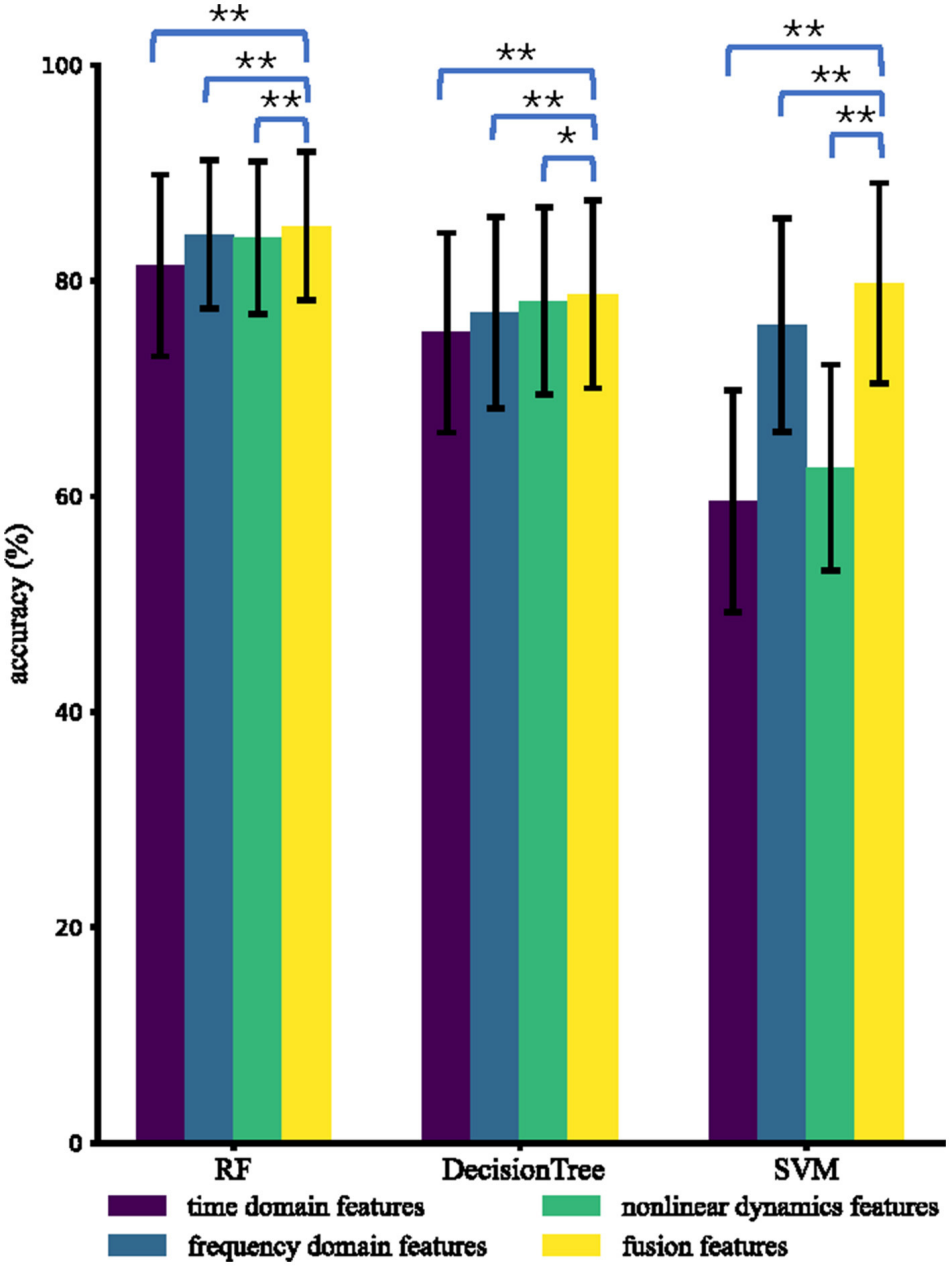
Average intra-subject accuracies using different types of features and different classifiers. The ^*^ and ^**^ indicate that the intra-subject accuracies of fusion features are significantly higher than those of the compared type of features with *p* < 0.05 and *p* < 0.01, respectively.

Figure 4 displays the mean and standard deviation of intra-subject accuracies for four types of features and three classifiers among 85 subjects. Fusion features demonstrate excellent performance, achieving accuracies (%) of 85.1, 78.7, and 79.8 using RF, DT, and SVM, respectively. For all classifiers (RF, DT, and SVM), the accuracies of fusion features are significantly greater than time domain features, frequency domain features, and non-linear dynamic features. RF performs the best among the three classifiers with different features, which is consistent with previous research findings that RF performs well on small datasets (Amin et al., 2017). The average accuracies (%) using RF are 81.4, 84.8, 84.0, and 85.1 for time domain features, frequency domain features, non-linear dynamic features, and fusion features, respectively.

### 3.2. Performance of inter-subject attention recognition

**Figure 5:**
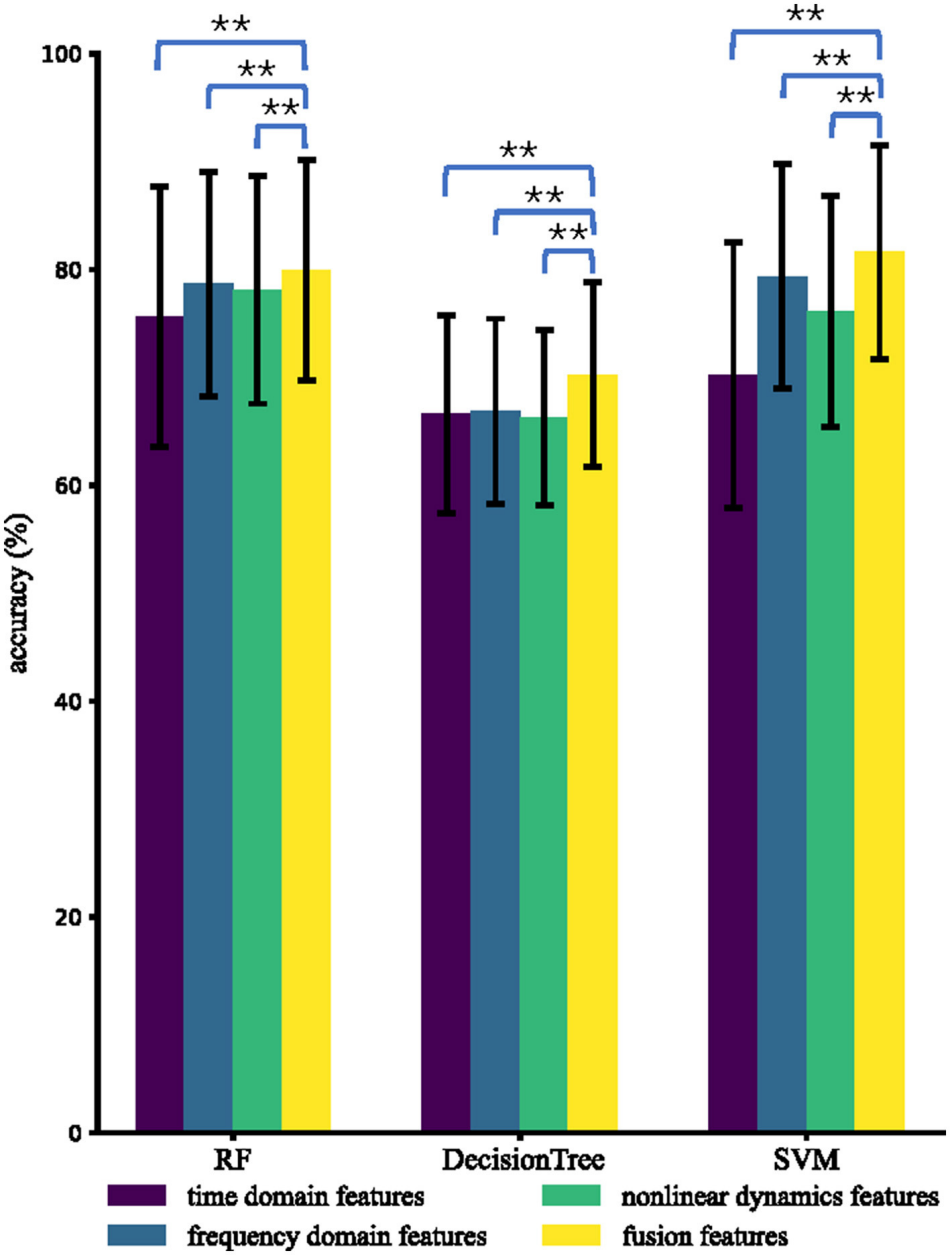
Average inter-subject accuracies using different types of features and different classifiers. The ^*^ and ^**^ indicate that the inter-subject accuracies of fusion features are significantly higher than those of the compared types of features with *p* < 0.05 and *p* < 0.01, respectively.

In terms of inter-subject analysis, we fixed features and classifiers as those in the intra-subject analysis. For each feature type and each classifier, we obtained 85 accuracies using LOSO CV and performed paired-sample *t*-tests to compare the accuracies of fusion features with those of other types of features. Figure 5 presents the inter-subject results obtained using different types of features and different classifiers, showing that RF and SVM were found to perform relatively well. Using RF, the average inter-subject accuracies (%) are 75.6, 78.7, 78.1, and 80.0 for the time domain features, frequency domain features, non-linear dynamic features, and fusion features, respectively. The best result (81.6%) of the average accuracies is achieved by SVM for distinguishing fusion features across subjects.

### 3.3. Assessment of our methods against baseline methods

Our study demonstrates the superior performance of our proposed method for the attention task, as compared to three baseline methods: PSD-SVM (Huang H. et al., 2021), Dynamical Complexity-XGBoost (Wan et al., 2021), and STFT-SVM (Acı et al., 2019). While PSD-SVM uses PSD features in the δ, θ, α, β, and γ bands and applies SVM for classification, Dynamical Complexity-XGBoost employs Multiscale Approximate Entropy, Sample Entropy, and Fuzzy Entropy as features and uses Extreme Gradient Boosting (XGBoost) for classification. Additionally, STFT-SVM utilizes the short-time Fourier transform (STFT) with a Blackman window to calculate smoothed time-dependent power spectra as features, which are then classified using SVM.

Our methods surpass the performance of baseline methods in both intra-subject and inter-subject classification, as evidenced by the results presented in Table 2. These findings demonstrate that our methods outperform the baseline methods.

**Table 2:** Average intra-subject and inter-subject accuracies using our methods (Fusion Feature-RF and Fusion Feature-SVM), PSD-SVM, Dynamical Complexity-XGBoost, and STFT-SVM.

**Intra-subject**	**Fusion feature-RF**	**PSD-SVM**	**Dynamical complexity-XGBoost**	**STFT-SVM**
Mean(%) ± std(%)	**85.05** **±** **6.87**	63.36 ± 9.64^**^	77.25 ± 11.51^**^	68.84 ± 9.68^**^
Inter-subject	Fusion Feature-SVM	PSD-SVM	Dynamical Complexity-XGBoost	STFT-SVM
Mean(%) ± std(%)	**81.60** **±** **9.93**	74.48 ± 13.36^**^	69.38 ± 11.98^**^	65.08± 10.62^**^

The ^*^ and ^**^ indicate that the accuracies of our methods are significantly higher than baseline methods in intra-subject or inter-subject classification with *p* < 0.05 and *p* < 0.01, respectively. Bold values indicate the superiority of our method over the basic method.

### 3.4. Individual feature analysis and classification performance evaluation

**Figure 6:**
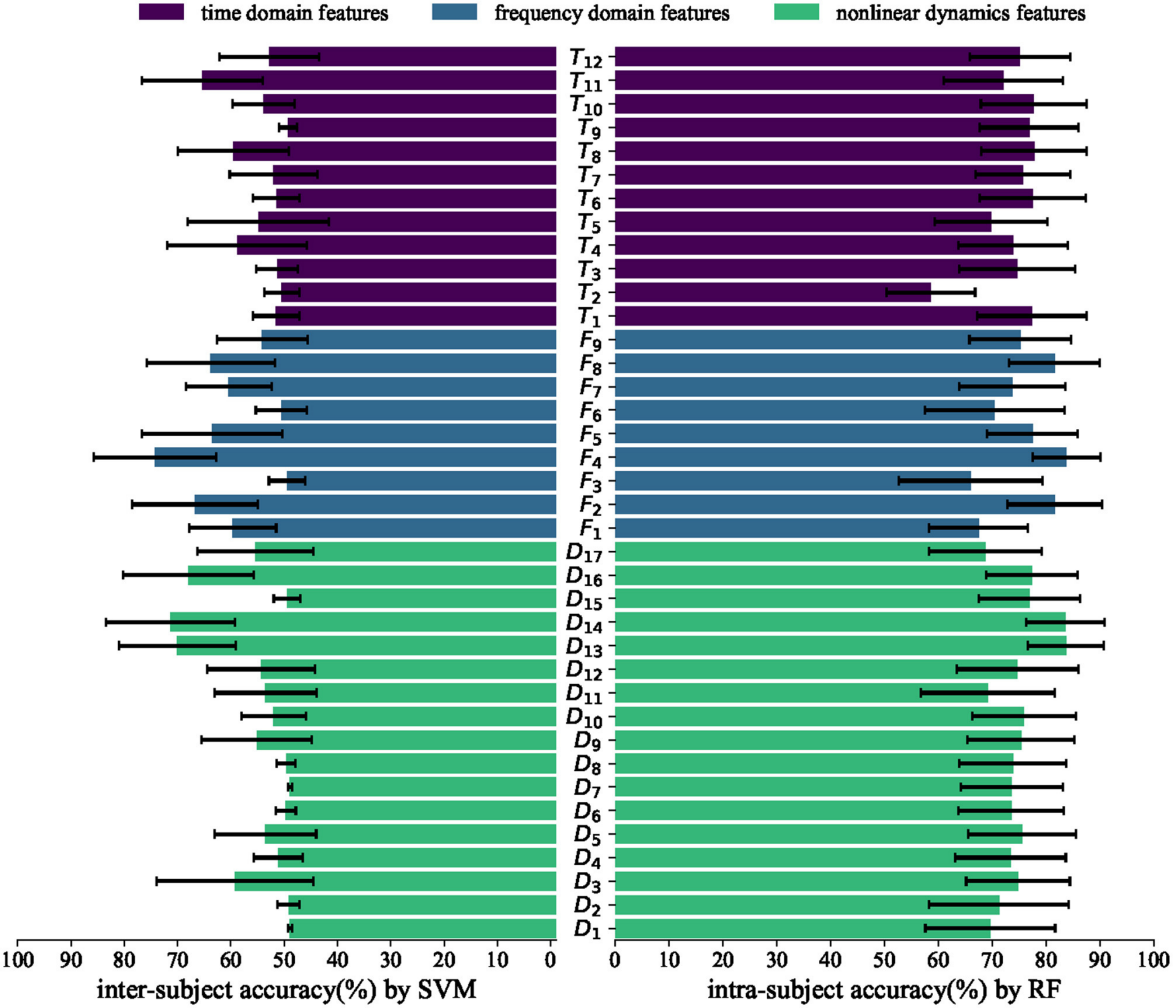
Average intra-subject accuracies by RF and inter-subject accuracies by SVM in different features. The horizontal axis represents accuracy, while the vertical axis represents different features.

To determine the effectiveness of features, we conducted an individual feature analysis by evaluating the classification performance. The results were calculated for intra-subject classification by RF and inter-subject classification by SVM, as depicted in Figure 6.

In the intra-subject experiment, numerous time domain, frequency domain, and non-linear dynamic features demonstrate an accuracy exceeding 70%. This finding suggests that the majority of the features calculated in Section 2 are effective within subjects. In the inter-subject experiment, only a few features exhibit a accuracy exceeding 70%, such as power spectrum for frequency bands *F*_4_, wavelet entropy *D*_13_, and Teager–kaiser energy *D*_14_. These features demonstrate robustness across subjects, indicating their effectiveness as more reliable features. The highest inter-subject accuracy (%) achieved by an individual feature is 75.24 ± 11.53. However, our proposed fusion feature demonstrates a significantly higher accuracy (%) of 81.60 ± 9.93. This result further confirms the effectiveness of our fusion feature method.

### 3.5. Neural patterns

First, we classified PSD features from different frequency bands (δ, θ, α, β, and γ) within and across subjects, respectively. The inter-subject and intra-subject average accuracies with features from different frequency bands are shown in Tables 3, 4, respectively. The average accuracies in Tables 3, 4 indicate that the PSD calculated from δ, θ, α, β, and γ bands exhibits distinct separability, which suggests that δ, θ, α, β, and γ oscillations of brain activity are related to the processing of attention states (Brown, 1970; Ray and Cole, 1985; Klimesch et al., 1993; Klimesch, 1999; Prinzel et al., 2001).

**Table 3:** Average intra-subject accuracies (%) of three classifiers for features from different frequency bands.

**Frequency bands**	**RF**	**DT**	**SVM**
Delta (0.5–4 Hz)	79.62	74.06	67.73
Theta (4–8 Hz)	80.78	76.28	68.00
Alpha (8–13 Hz)	81.53	75.08	67.84
Beta (13–30 Hz)	80.76	74.28	65.58
Gamma (30–50 Hz)	80.28	74.93	64.53

**Table 4:** Average inter-subject accuracies (%) of three classifiers for features from different frequency bands.

**Frequency bands**	**RF**	**DT**	**SVM**
Delta (0.5–4 Hz)	70.82	62.17	63.90
Theta (4–8 Hz)	69.38	60.62	62.71
Alpha (8–13 Hz)	70.57	60.99	62.71
Beta (13–30 Hz)	73.83	64.60	63.99
Gamma (30–50 Hz)	74.99	65.06	65.35

**Figure 7:**
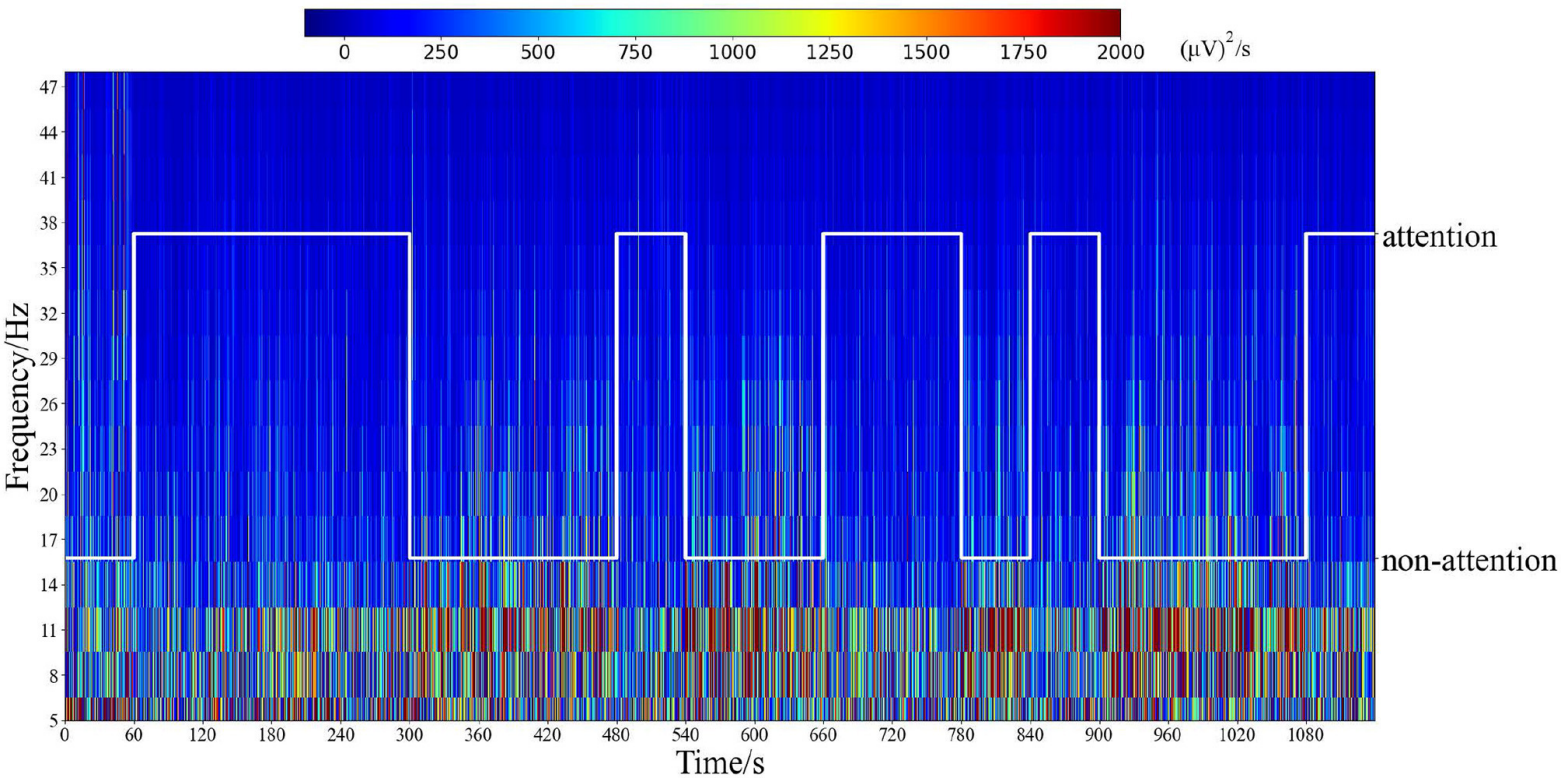
Time–frequency analysis by Morlet wavelets of the electrode position Fz in all epochs for one subject. The white line represents the labels of attention states corresponding to time.

Additionally, time–frequency analysis with Morlet wavelets Cohen (2019) was employed in electrode position Fz in an experiment, as depicted in Figure 7. This figure illustrates the distinct patterns observed for different attention states. Notably, the analysis reveals that frequencies below 30 Hz exhibit significantly lower response energy during the attention state compared to the non-attention state. These findings further support the relationship between attention states and frequency bands such as α, β, and θ.

**Figure 8:**
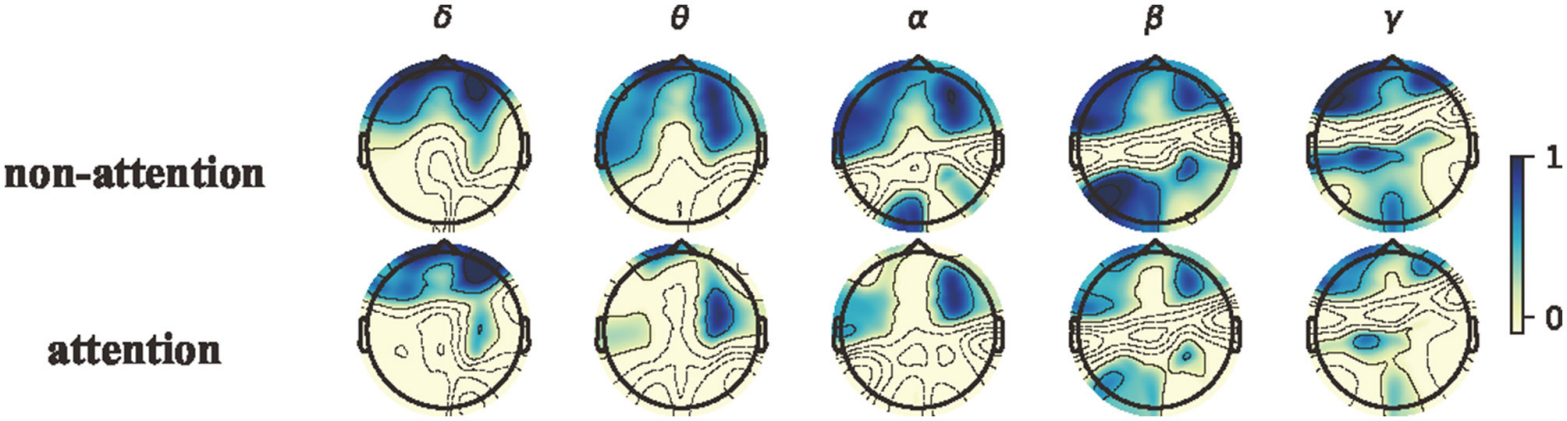
Topographical maps of power features in the canonical frequency bands. From left to right: δ, θ, α, β, and γ bands; from top to bottom: non-attention and attention.

To further explore neural patterns associated with attention and non-attention states across all participants, we calculated the topographical maps of power features by averaging the power features over all epochs in all subjects for each frequency band between attention states and non-attention states. We then normalized the features by Z-Score for all epochs within each frequency band for each subject. Figure 8 depicts the topographical maps of the power features corresponding to the attention state an the non-attention state. The results demonstrate the existence of neural patterns associated with attention and non-attention states.

Although the neural patterns of attention states and non-attention states are similar, greater activation iobserved in the prefrontal areas for both. In the α, β, and θ bands, the lateral prefrontal and occipital areas exhibit less activation during attention states than non-attention states. During non-attention states, there are significant higher β responses in both prefrontal and occipital regions. The existing studies (Klimesch et al., 1998; Egner and Gruzelier, 2004) have showed that the changes in EEG features are closely related to the degree of attention, with varying degrees of amplitude and power of individual rhythmic brain waves. For instance, when participants were in the state of attention, their EEG signals exhibited a significant decrease in α and β waves (Prinzel et al., 2001). Conversely, during non-attention processing, the energy of β and α responses was increased. These findings on neural patterns are consistent with previous attention studies (Ray and Cole, 1985; Klimesch, 1999; Kelly et al., 2003; Swartwood et al., 2003; K Binienda et al., 2011).

## 4. Discussion

In this study, we first designed a novel attention experiment paradigm and collected a dataset consisting of 85 subjects. Next, we extracted and fused time domain, frequency domain, and non-linear dynamic features. These features were then classified to construct a complete attention recognition framework. Additionally, we suggested that conducting a separate analysis of the differences in features and channels at the group level may be useful in distinguishing between attention and non-attention states.

The following discussions will be divided into six parts. First, we compared and analyzed the different features in different channels of attention and non-attention states, which is useful in the construction of our attention recognition framework. Second, we conducted a group level analysis on connectivity estimators of the attention and non-attention states. Third, we also discussed the neural patterns of attention and non-attention states. Fourth, we analyzed and compared different paradigms for attention recognition. Fifth, we conducted an advantages analysis on our method. Last, we described the limitations of this study and future research perspectives.

### 4.1. A group level analysis on the features of the attention and non-attention states

In our study, we conducted an analysis of the differences in the different types of features across channels between attention and non-attention states at the group level. Specifically, we collected features extracted from each channel of different epochs for each subject. We then divided the features into two parts for attention and non-attention states, respectively. After removing outliers, we averaged each part to obtain the average features, which represented the average level of a specific feature for a given channel and subject for attention and non-attention states.

**Figure 9:**
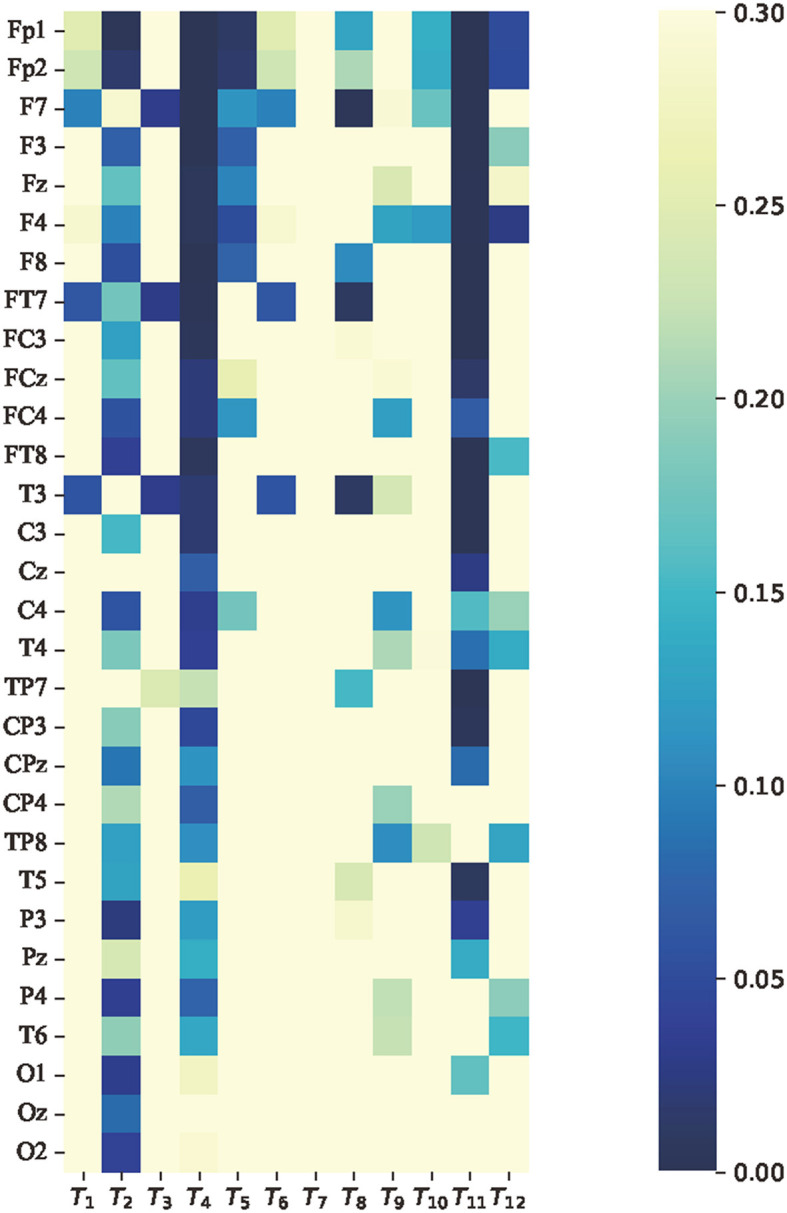
*p*-value of average features of time domain features paired *t*-test between attention and non-attention. The vertical axis represents 30 channels. From top to bottom, the channel names are as follows: Fp1, Fp2, F7, F3, Fz, F4, F8, FT7, FC3, FCz, FC4, FT8, T7, C3, Cz, C4, T8, TP7, CP3, CPz, CP4, TP8, P7, P3, Pz, P4, P8, O1, Oz, and O2. The horizontal axis represents the 24 dimensional time domain features, which are consistent with Table 1.

**Figure 10:**
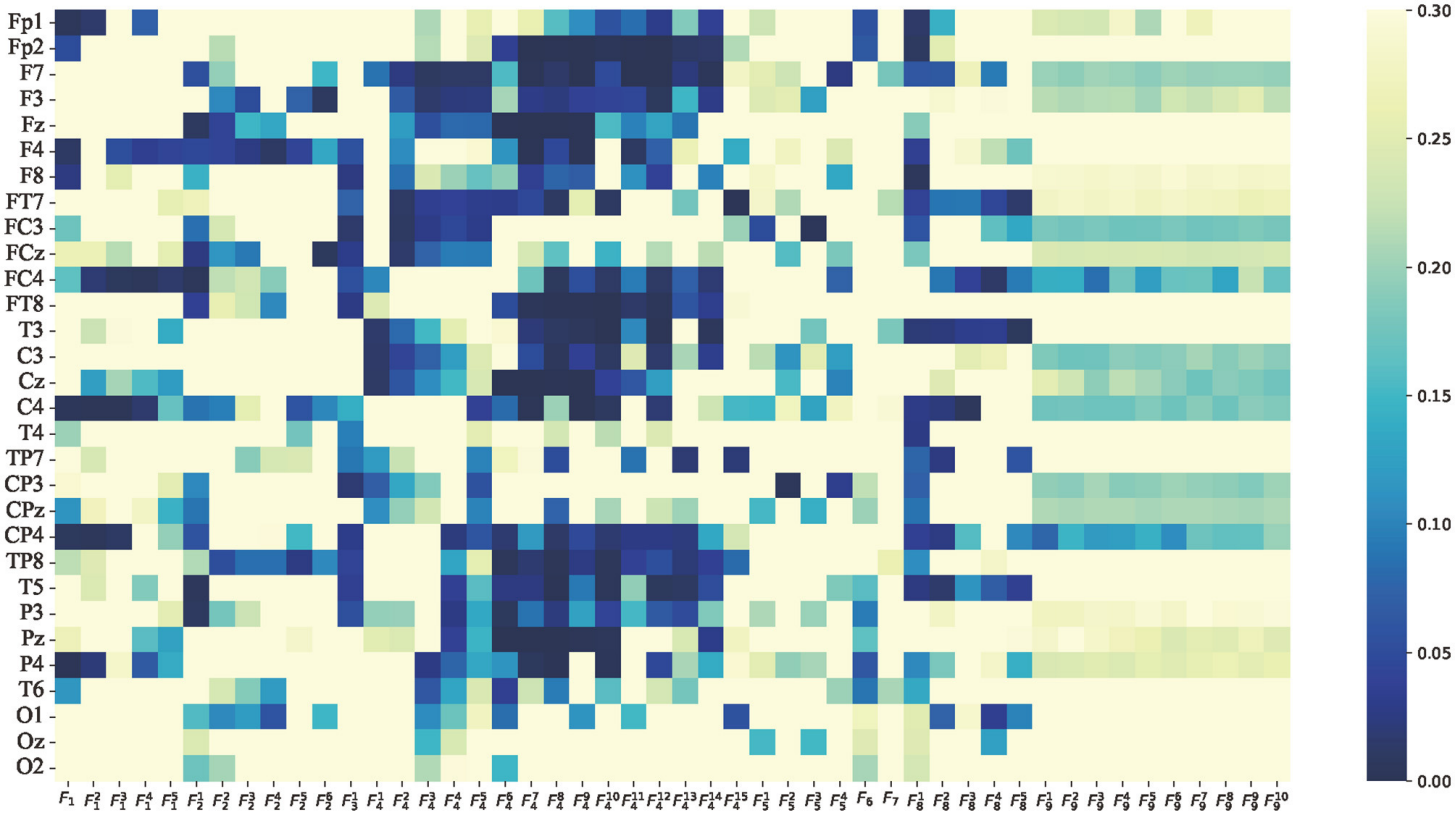
*p*-value of average features of frequency domain features paired *t*-test between attention and non-attention. The vertical axis is the same as Figure 9. The horizontal axis represents the 48-dimensional frequency domain features, which is consistent with Table 1. The 14-dimensional PSD features *F*_4_ represent δ, θ, α, β, γ, δ/θ, δ/α, δ/β, δ/γ, θ/α, θ/β, θ/γ, α/β, α/γ, and β/γ from left to right.

**Figure 11:**
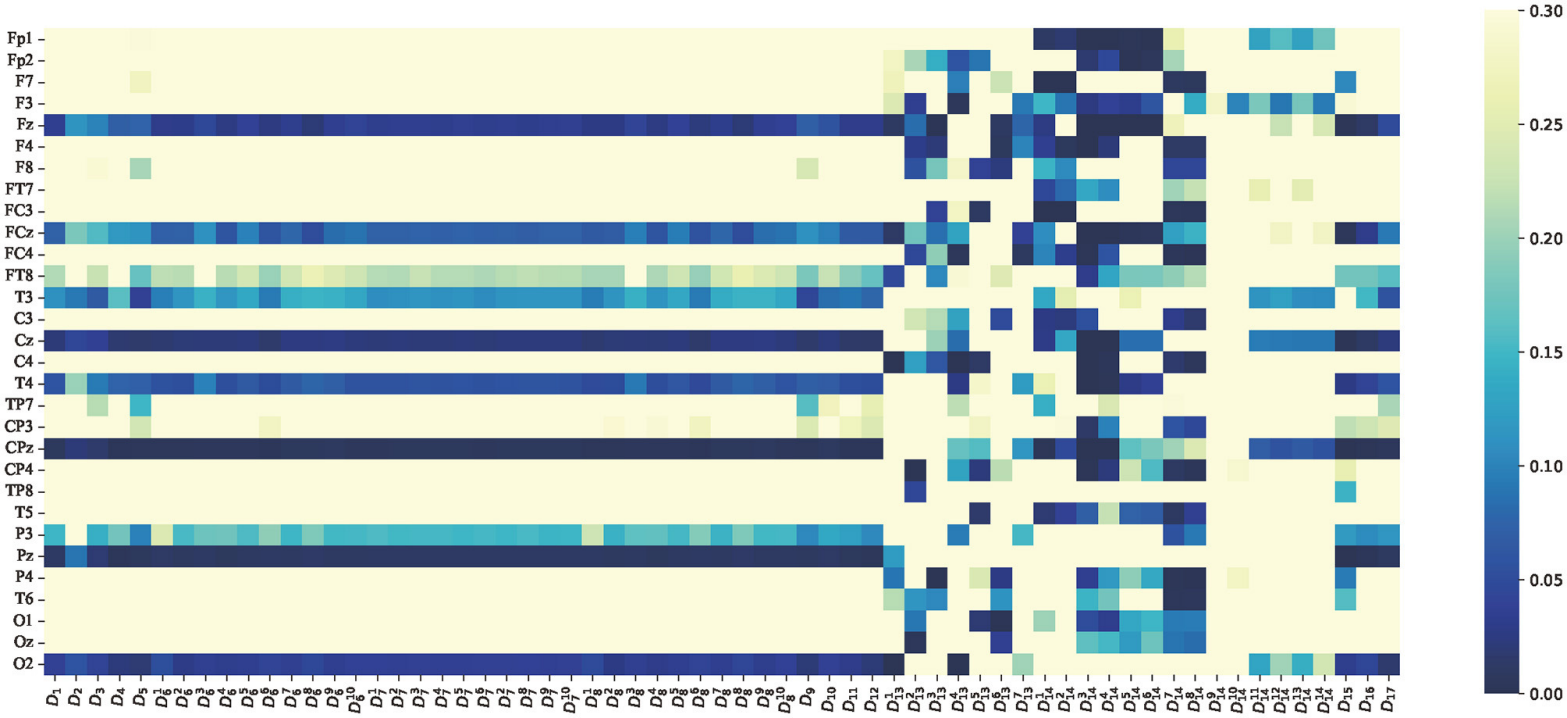
*p*-value of average features of non-linear dynamic features paired *t*-test between attention and non-attention. The vertical axis is the same as Figure 9. The horizontal axis represents the 60 dimensional non-linear dynamic features, which are consistent with Table 1.

To analyze whether there were significant differences in the average features between attention and non-attention states at the group level, paired *t*-tests were implemented on the average features in the time domain, frequency domain, and non-linear dynamic features, respectively. It is assumed that the average features of attention and non-attention do not significantly differ at the group level. The *p*-value of 0.05 is used as a significance level. This means that if the *p*>0.05, the null hypothesis is true; otherwise false. The results of the *t*-tests in terms of time domain features, frequency domain features, and non-linear dynamic features are, respectively, shown in Figures 9–11.

Regarding the average features of time domain features, it was found that for most of the channels corresponding to the feature skewness *T*_4_ and decorrelation time *T*_11_, the *p*-values were much less than 0.05, indicating a relatively significant difference. This finding is consistent with the results of previous studies that have investigated the relationship between skewness and cognition in EEG signals Davis et al. (2020). Additionally, Figure 9 shows that prefrontal channels such as Fp1, Fp2, F7, F3, Fz, and F4 exhibit more significant features relative to other channels.

Compared to the average features of time domain features, the average features of frequency domain features performed better in the *t*-test. The *p*-values of the average features of *F*_4_ (power spectrum density) of θ, α, β, and power spectral density ratio δ/γ, δ/θ, δ/α, δ/β, δ/γ, θ/α, θ/β, θ/γ, α/β, and α/γ were less than 0.05 in frontal, occipital, and temporal brain regions, as shown in Figure 10. These significant differences at the group level explain the superior performance of frequency domain features compared to time domain features in Figures 4, 5. This finding is consistent with previous studies that cognitive tasks can enhance the power of eeg, particularly in visual cortex Fitzgibbon et al. (2004).

As indicated in Figure 11, the average features of non-linear dynamic features are very special, and most of the non-linear features are significant for part of the channels. This finding is consistent with previous attention studies that have found that the value of entropy decreases with a decrease of attention states (Li et al., 2012). For instance, the features *D*_14_ (Teager–kaiser energy) exhibit a highly significant difference between attention and non-attention states, as shown in Figure 11, which indicates that Teager–kaiser energy *D*_14_ is an excellent feature. Furthermore, the first eight dimensions of Teager–kaiser energy exhibit more significant channels and a deeper degree than the last six dimensions. According to the content in Section 2, the first eight dimensions correspond to Teager–kaiser energy of the low-frequency portion of wavelet decomposition, while the last six dimensions correspond to Teager–kaiser energy of the high-frequency portion of wavelet decomposition. This highlights that the contrast between attention and non-attention states in low-frequency portion is much more pronounced than in high-frequency portion, which is consistent with previous research (Fiebelkorn and Kastner, 2019).

Taken together, some average features from time domain, frequency domain, and non-linear dynamics exhibit significant differences at the group level, which further validates the effectiveness of our framework for attention recognition based on EEG.

### 4.2. A group level analysis on connectivity estimators of the attention and non-attention states

**Figure 12:**
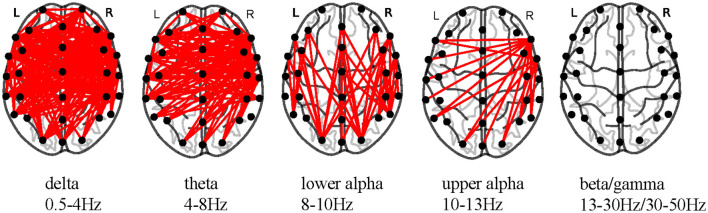
Significant differences in FC estimators across frequency bands (*p* < 0.05, FDR-corrected).

Brain functional connectivity (FC) elucidating the statistical dependencies and directed information flows unveils the functions and intricate interactions of diverse brain regions (Cao et al., 2022). We estimated correlations between different channels for each epoch of each participant to construct FC matrices and separately averaged the FC matrices of attention and non-attention states for each participant. This allowed us to obtain the average FC matrices for attention and non-attention states. We then conducted paired-sample *t*-tests and corrected using the false discovery rate (FDR) method for the average FC matrices corresponding to the two states. Figure 12 shows significant differences in brain FCs between attention and non-attention states across different frequency bands at the group level.

From Figure 12, it is evident that the significant differences in brain FCs between attention and non-attention states are primarily concentrated in the low-frequency bands, including δ, θ, lower α, and upper α (Zoefel et al., 2011) frequency bands. Within δ and θ frequency bands, there are significantly different connectivities distributed in various brain regions, including the left and right temporal lobes, parietal lobes, prefrontal regions, and occipital lobes. Within lower α and upper α frequency bands, the connectivities are mainly concentrated in the left and right temporal lobes. Overall, as the frequency range increases, there is a decrease in the number of significant connectivities, which further demonstrates that lower frequencies are more capable of characterizing changes in attention states. This is consistent with our discussion in section 4.1.

Furthermore, by comparing the differences in relatively low-frequency connectivities between the left and right hemispheres, we found that the number of significant connectivities in the right hemisphere at the group level was significantly higher than that in the left hemisphere. This indicates that the right hemisphere interacts more closely with information during attention changes, which is consistent with previous studies showing significant hemispheric asymmetry and lateralization toward the right hemisphere in the attention process of individuals (Bartolomeo and Malkinson, 2019).

### 4.3. Neural patterns analysis on the attention and non-attention states

In Section 3, we observed that neural patterns for attention and non-attention states exist according to Tables 3, 4, Figure 8. In this section, we analyzed these neural patterns of attention and non-attention states in greater detail.

**Figure 13:**
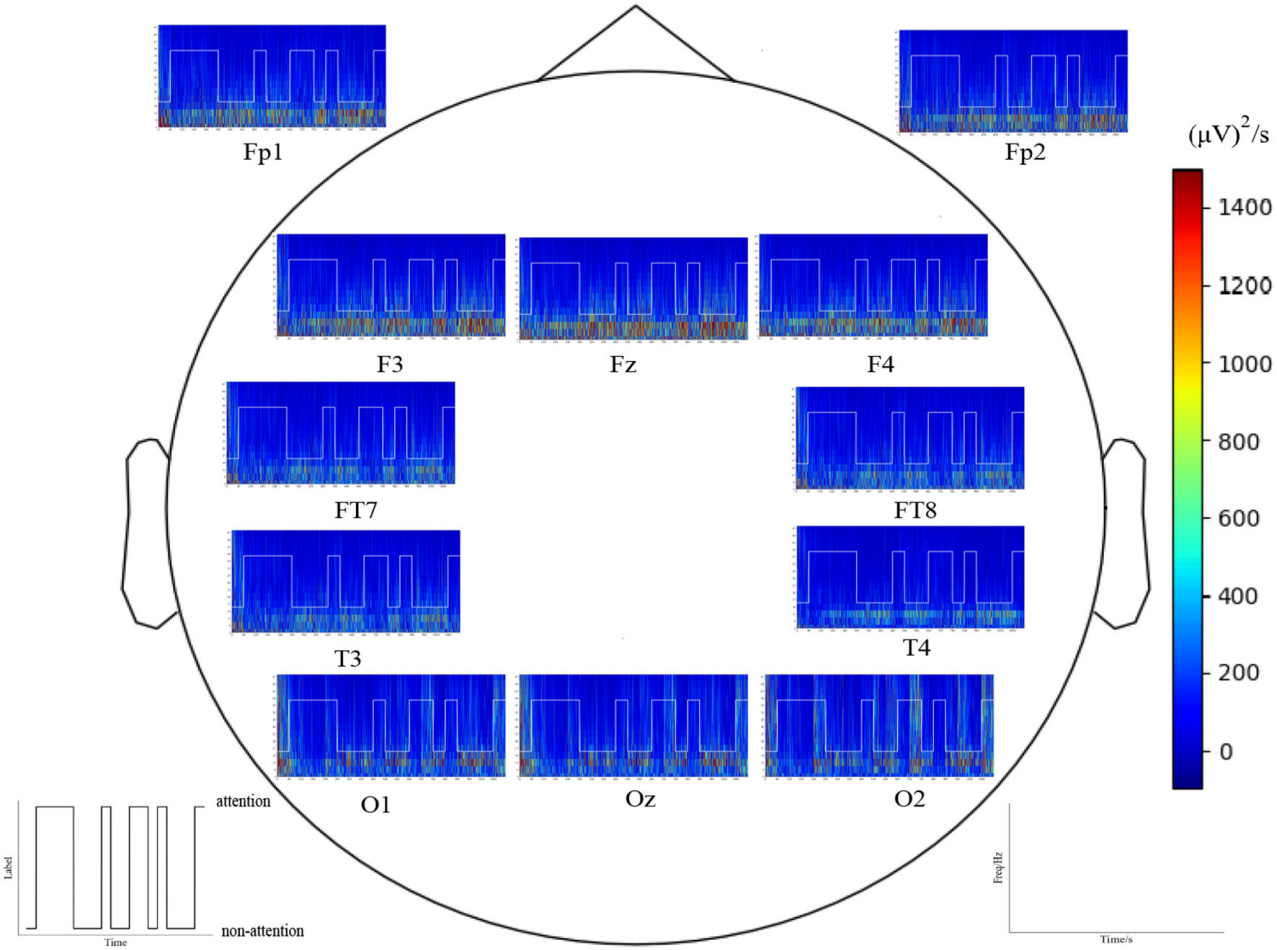
Time–frequency analysis by Morlet wavelets of the electrode position Fp1, Fp2, Fz, F3, F4, T3, T4, FT7, FT8, O1, Oz, and O2 in all epochs for one subject a red color indicates a high amplitude).

Figure 13 shows the time–frequency analysis using Morlet wavelets for all epochs recorded from electrodes Fp1, Fp2, Fz, F3, F4, T3, T4, FT7, FT8, O1, Oz, and O2 in an experiment. As demonstrated by Figures 7 and 13, the time–frequency analysis reveals different patterns for different attention states. Specifically, the responses of low-frequency oscillations during attention states are lower than during non-attention states, especially in the temporal lobes and prefrontal regions. Additionally, the neural patterns remain relatively stable over time for each epoch within the experiment.

As shown in Figure 8, the average PSD in the θ, α, and β bands across all subjects exhibits distinct differences between attention and non-attention states, whereas this in the δ do not. This implies that the 4–30 Hz frequency bands are more closely associated with attention than the other frequency bands in EEG signals, which is consistent with prior research (Prinzel et al., 2001). For the δ band, compared with non-attention states, the neural patterns of attention states have a significantly lower response in the prefrontal. For the θ, β, and α bands, compared with non-attention states, the neural patterns of attention states have a significantly lower response in the lateral prefrontal and occipital areas.

### 4.4. A comparative analysis on paradigms

As described in Section 1, the tasks in paradigms of attention recognition include the Stroop test, breath counting, and reading comprehension. The Stroop test is inherently short in duration (Kawashima et al., 2023), which makes it difficult for participants to sustain their attention over time. The task of counting the number of breaths can easily lead to mental wandering (Braboszcz and Delorme, 2011). Additionally, reading tasks are influenced by different materials, leading to variations in attention and concentration levels among individuals (Li et al., 2011). To address these limitations, we proposed mental arithmetic tasks as an attention task in our paradigm. Mental arithmetic tasks have a longer duration and are not affected by different materials. Our framework's results provide strong evidence for the effective characterization of attention and non-attention states under our proposed paradigm. First, the excellent classification results in Figures 4, 5 demonstrate that attention states and non-attention states can be distinguished accurately with our paradigm. Second, different activation maps in Figure 8 between attention and non-attention states further support the efficacy of our proposed paradigm in characterizing attention and non-attention states. Together, these findings highlight the potential value of our paradigm in advancing research and understanding of attention states.

### 4.5. An advantages analysis on our method

First, our method can not only be used for intra-subject attention recognition but also for inter-subject attention recognition, making the application more convenient. Second, we can see that our proposed fusion feature method achieves the accuracy (%) of 85.05 ± 6.87 in intra-subject attention recognition and 81.60 ± 9.93 in inter-subject attention recognition. Compared with other attention classification methods, it achieves better classification results both within and across subjects, making the results of attention recognition more accurate.

### 4.6. Limitations and future study

There are three limitations to this study. First, this study analyzed the EEG features between attention states and non-attention states offline and did not perform some online validation. Thus, we will perform online validation for the framework presented in this study in future article. Second, in this study, all EEG channels (30 channels) were used to calculate the features, and it is difficult to collect 30 channels of EEG data for the complex variety of application scenarios of attention recognition (such as hospitals and schools). Therefore, reducing the number of channels for attention recognition is also an important direction for our future study. Third, in this study, for attention and non-attention states, we analyzed and classified the manual features based on EEG wthin and across subjects, and it is necessary to design an end-to-end network framework on large dataset in future study.

## 5. Conclusion

Attention recognition is of great importance in various fields such as medicine and industry. However, a reliable inter-subject attention recognition framework that can be effective is still missing. This study proposed a novel attention experiment paradigm, built a dataset of 85 subjects, fused three types of features, and classified features for attention recognition based on EEG. Eighty-five subjects participated in our experiment, and the experimental results demonstrated the validity of our paradigm and analysis methods with an average intra-subject attention recognition accuracy of 85.05% and an average inter-subject attention recognition accuracy of 81.60%. Additionally, our frequency band features analysis revealed neural patterns of attention and non-attention states, where attention states showed less activation than non-attention states in the prefrontal and occipital areas in α, β, and θ bands. Furthermore, we identified the features that exhibited signification corresponding channels between attention and non-attention states. These findings may be useful for understanding attention recognition based on EEG and may guide future study in this area.

## Data availability statement

The raw data supporting the conclusions of this article will be made available by the authors, without undue reservation.

## Ethics statement

The studies involving human participants were reviewed and approved by Ethics Committee of Affiliated Brain Hospital of Guangzhou Medical University, Guangzhou, China. The patients/participants provided their written informed consent to participate in this study. Written informed consent was obtained from the individual(s) for the publication of any potentially identifiable images or data included in this article.

## Author contributions

HH and YL built the attention paradigm and did the experiment in this study. DC did the study analysis and wrote the study with help from HH, XB, JP, and YL. All authors contributed to the article and approved the submitted version.

## References

[B1] Acı,¸ C. İ. Kaya M. Mishchenko Y. (2019). Distinguishing mental attention states of humans via an EEG-based passive BCI using machine learning methods. Expert Syst. Appl. 134, 153–166. 10.1016/j.eswa.2019.05.05736749989

[B2] Amin H. U. Mumtaz W. Subhani A. R. Saad M. N. M. Malik A. S. (2017). Classification of EEG signals based on pattern recognition approach. Front. Comput. Neurosci. 11, 103. 10.3389/fncom.2017.00103 29209190 PMC5702353

[B3] Andrillon T. Burns A. Mackay T. Windt J. Tsuchiya N. (2021). Predicting lapses of attention with sleep-like slow waves. Nat. Commun. 12, 3657. 10.1038/s41467-021-23890-7 34188023 PMC8241869

[B4] Badani S. Saha S. Kumar A. Chatterjee S. Bose R. (2017). “Detection of epilepsy based on discrete wavelet transform and teagerkaiser energy operator,” in 2017 IEEE Calcutta Conference (CALCON). Kolkata: IEEE, 164-167. 10.1109/CALCON.2017.828071734607322

[B5] Bai D. Qiu T. Li X. (2007). The sample entropy and its application in EEG based epilepsy detection. J. Biomed. Eng. 24, 200–205. 17333922

[B6] Barry R. J. Kirkaikul S. Hodder D. (2000). EEG alpha activity and the ERP to target stimuli in an auditory oddball paradigm. Int. J. Psychophysiol. 39, 39–50. 10.1016/S0167-8760(00)00114-8 11120346

[B7] Bartolomeo P. Malkinson T. S. (2019). Hemispheric lateralization of attention processes in the human brain. Curr. Opin. Psychol. 29, 90–96. 10.1016/j.copsyc.2018.12.023 30711910

[B8] Berka C. Levendowski D. J. Cvetinovic M. M. Petrovic M. M. Davis G. Lumicao M. N.. (2004). Real-time analysis of EEG indexes of alertness, cognition, and memory acquired with a wireless EEG headset. Int. J. Hum. Comput. Interact. 17:151–170. 10.1207/s15327590ijhc1702_3

[B9] Blanco S. Figliola A. Quiroga R. Q. Rosso O. Serrano E. (1998). Time-frequency analysis of electroencephalogram series. III. wavelet packets and information cost function. Phys. Rev. E 57, 932. 10.1103/PhysRevE.57.932

[B10] Borbely A. A. Neuhaus H. U. (1979). Sleep-deprivation: effects on sleep and EEG in the rat. J. Comparat. Physiol. 133, 71–87. 10.1007/BF0066311110081927

[B11] Braboszcz C. Delorme A. (2011). Lost in thoughts: neural markers of low alertness during mind wandering. Neuroimage. 54, 3040–3047. 10.1016/j.neuroimage.2010.10.008 20946963

[B12] Brown B. B. (1970). Recognition of aspects of consciousness through association with EEG alpha activity represented by a light signal. Psychophysiology. 6, 442–452. 10.1111/j.1469-8986.1970.tb01754.x 5418811

[B13] Cai H. Qu Z. Li Z. Zhang Y. Hu X. Hu B. (2020). Feature-level fusion approaches based on multimodal eeg data for depression recognition. Informat. Fusi. 59, 127–138. 10.1016/j.inffus.2020.01.008

[B14] Candra H. Yuwono M. Handojoseno A. Chai R. Su S. Nguyen H. T. (2015). “Recognizing emotions from EEG subbands using wavelet analysis,” in 2015 37th Annual International Conference of the IEEE Engineering in Medicine and Biology Society (EMBC). Milano: IEEE, 6030–6033. 10.1109/EMBC.2015.7319766 26737666

[B15] Cao J. Zhao Y. Shan X. Wei H.-,l. Guo Y. Chen L.. (2022). Brain functional and effective connectivity based on electroencephalography recordings: a review. Hum. Brain Mapp. 43, 860–879. 10.1002/hbm.25683 34668603 PMC8720201

[B16] Capurro A. Diambra L. Lorenzo D. Macadar O. Martín M. T. Mostaccio C.. (1998). Tsallis entropy and cortical dynamics: the analysis of EEG signals. Physica A. 257, 149–155. 10.1016/S0378-4371(98)00137-X

[B17] Chen H. Song Y. Li X. (2019). Use of deep learning to detect personalized spatial-frequency abnormalities in eegs of children with ADHD. J. Neural Eng. 16, 066046. 10.1088/1741-2552/ab3a0a 31398717

[B18] Chin Y. J. Ong T. S. Teoh A. B. J. Goh K. (2014). Integrated biometrics template protection technique based on fingerprint and palmprint feature-level fusion. Informat. Fusion. 18, 161–174. 10.1016/j.inffus.2013.09.001

[B19] Chin Z. Y. Zhang X. Wang C. Ang K. K. (2018). “EEG-based discrimination of different cognitive workload levels from mental arithmetic,” in 2018 40th Annual International Conference of the IEEE Engineering in Medicine and Biology Society (EMBC). Honolulu, HI: IEEE, 1984–1987. 10.1109/EMBC.2018.8512675 30440788

[B20] Chun M. M. Golomb J. D. Turk-Browne N. B. (2011). A taxonomy of external and internal attention. Annu. Rev. Psychol. 62, 73–101. 10.1146/annurev.psych.093008.100427 19575619

[B21] Cohen M. X. (2019). A better way to define and describe morlet wavelets for time-frequency analysis. Neuroimage. 199, 81–86. 10.1016/j.neuroimage.2019.05.048 31145982

[B22] Conners C. K. Sitarenios G. Parker J. D. Epstein J. N. (1998). The revised Conners' Parent Rating Scale (CPRS-R): factor structure, reliability, and criterion validity. J. Abnorm. Child Psychol. 26, 257–268. 10.1023/A:1022602400621 9700518

[B23] Davis J. J. Schbeler F. Ji S. Kozma R. (2020). Discrimination between brain cognitive states using shannon entropy and skewness information measure, in *2020 IEEE International Conference on Systems, Man, and Cybernetics (SMC)*. Toronto, ON: IEEE, 4026–4031. 10.1109/SMC42975.2020.9283315

[B24] Delorme A. Makeig S. Sejnowski T. (2001). “Automatic artifact rejection for EEG data using high-order statistics and independent component analysis,” in Proceedings of the Third International ICA Conference. Princeton: Citeseer, 9–12.

[B25] Demanuele C. James C. J. Sonuga-Barke E. J. (2007). Distinguishing low frequency oscillations within the 1/f spectral behaviour of electromagnetic brain signals. Behav. Brain Funct. 3, 1–14. 10.1186/1744-9081-3-62 18070337 PMC2235870

[B26] Dumermuth G. Molinari L. (1987). Spectral analysis of the EEG. Neuropsychobiology. 17, 85–99. 10.1159/000118345 3306442

[B27] Egner T. Gruzelier J. H. (2004). EEG biofeedback of low beta band components: frequency-specific effects on variables of attention and event-related brain potentials. Clini. Neurophysiol. 115, 131–139. 10.1016/S1388-2457(03)00353-5 14706480

[B28] Esteller R. Echauz J. Tcheng T. Litt B. Pless B. (2001). “Line length: an efficient feature for seizure onset detection,” in 2001 Conference Proceedings of the 23rd Annual International Conference of the IEEE Engineering in Medicine and Biology Society, volume 2. Istanbul: IEEE, 1707–1710. 10.1109/IEMBS.2001.102054517442001

[B29] Esteller R. Vachtsevanos G. Echauz J. Lilt B. (1999). “A comparison of fractal dimension algorithms using synthetic and experimental data,” in 1999 IEEE International Symposium on Circuits and Systems (ISCAS). Orlando, FL: IEEE, 199–202. 10.1109/ISCAS.1999.778819

[B30] Fiebelkorn I. C. Kastner S. (2019). A rhythmic theory of attention. Trends Cogn. Sci. 23, 87–101. 10.1016/j.tics.2018.11.009 30591373 PMC6343831

[B31] Fitzgibbon S. Pope K. Mackenzie L. Clark C. Willoughby J. (2004). Cognitive tasks augment gamma EEG power. Clini. Neurophysiol. 115, 1802–1809. 10.1016/j.clinph.2004.03.009 15261859

[B32] Fliege H. Becker J. Walter O. B. Rose M. Bjorner J. B. Klapp B. F. (2009). Evaluation of a computer adaptive test for the assessment of depression (D'CAT) in clinical application. Int. J. Methods Psychiatr. Res. 18, 23–36. 10.1002/mpr.274 19194856 PMC6878570

[B33] Gao W. Yu T. Yu J.-G. Gu Z. Li K. Huang Y.. (2021). Learning invariant patterns based on a convolutional neural network and big electroencephalography data for subject-independent P300 brain-computer interfaces. IEEE Trans. Neural Syst. Rehabil. Eng. 29, 1047–1057. 10.1109/TNSRE.2021.3083548 34033543

[B34] Grabner R. H. De Smedt B. (2011). Neurophysiological evidence for the validity of verbal strategy reports in mental arithmetic. Biol. Psychol. 87, 128–136. 10.1016/j.biopsycho.2011.02.019 21382434

[B35] Grieszbach G. Schack B. (1993). Adaptive quantile estimation and its application in analysis of biological signals. Biom. J. 35, 165–179. 10.1002/bimj.471035020720205787

[B36] Gudmundsson S. Runarsson T. P. Sigurdsson S. (2005). “Automatic sleep staging using support vector machines with posterior probability estimates,” in International Conference on Computational Intelligence for Modelling, Control and Automation and International Conference on Intelligent Agents, Web Technologies and Internet Commerce (CIMCA-IAWTIC'06), volume 2. Vienna: IEEE, 366–372. 10.1109/CIMCA.2005.1631496

[B37] Hamadicharef B. Zhang H. Guan C. Wang C. Phua K. S. Tee K. P.. (2009). “Learning EEG-based spectral-spatial patterns for attention level measurement,” in 2009 IEEE International Symposium on Circuits and Systems. Seoul: IEEE, 1465–1468. 10.1109/ISCAS.2009.5118043

[B38] Hester R. Garavan H. (2005). Working memory and executive function: the influence of content and load on the control of attention. Memory & *Cognit*. 33:221–233. 10.3758/BF03195311 16028577

[B39] Hjorth B. (1970). EEG analysis based on time domain properties. Electroencephalogr. Clin. Neurophysiol. 29, 306–310. 10.1016/0013-4694(70)90143-4 4195653

[B40] Hosseini S. Guo X. (2019). “Deep convolutional neural network for automated detection of mind wandering using EEG signals,” in Proceedings of the 10th ACM International Conference on Bioinformatics, Computational Biology and Health Informatics. New York: Association for Computing Machinery, 314–319. 10.1145/3307339.3342176

[B41] Hu B. Li X. Sun S. Ratcliffe M. (2016). Attention recognition in EEG-based affective learning research using CFS+KNN algorithm. IEEE/ACM Trans Comput Biol. Bioinform. 15, 38–45. 10.1109/TCBB.2016.2616395 27740494

[B42] Huang H. Cai Y. Feng X. Li Y. (2021). An electroencephalogram-based study of resting-state spectrogram and attention in tinnitus patients. J. Biomed. Eng. 38, 492–497. 10.7507/1001-5515.202012015 34180194 PMC9927779

[B43] Huang W. Wu W. Lucas M. V. Huang H. Wen Z. Li Y. (2021). “Neurofeedback training with an electroencephalogram-based brain-computer interface enhances emotion regulation,” in IEEE Transactions on Affective Computing (IEEE).

[B44] Inouye T. Shinosaki K. Sakamoto H. Toi S. Ukai S. Iyama A.. (1991). Quantification of EEG irregularity by use of the entropy of the power spectrum. Electroencephalogr. Clin. Neurophysiol. 79:204–210. 10.1016/0013-4694(91)90138-T 1714811

[B45] K Binienda Z. A. Beaudoin M. T. Thorn B.. (2011). Analysis of electrical brain waves in neurotoxicology: gamma-hydroxybutyrate. Curr. Neuropharmacol. 9:236–239. 10.2174/157015911795017209 21886596 PMC3137189

[B46] Kannathal N. Acharya U. R. Lim C. M. Sadasivan P. (2005). Characterization of EEG a comparative study. Comput. Methods Programs Biomed. 80, 17–23. 10.1016/j.cmpb.2005.06.005 16099533

[B47] Kawashima I. Nagahama T. Kumano H. Momose K. Tanaka S. C. (2023). Pavlovian-based neurofeedback enhances meta-awareness of mind-wandering. Neural Networks. 158, 239–248. 10.1016/j.neunet.2022.11.024 36473291

[B48] Ke Y. Chen L. Fu L. (2014). Visual attention recognition based on nonlinear dynamical parameters of EEG. Biomed. Mater. Eng. 24, 349–355. 10.3233/BME-130817 24211916

[B49] Kelly S. Dockree P. Reilly R. Robertson I. (2003). “EEG alpha power and coherence time courses in a sustained attention task,” in First International IEEE EMBS Conference on Neural Engineering, 2003. Conference Proceedings. Capri: IEEE, 83–86. 10.1109/CNE.2003.1196761

[B50] Kharbouch A. Shoeb A. Guttag J. Cash S. S. (2011). An algorithm for seizure onset detection using intracranial EEG. Epilepsy Behav. 22, S29–S35. 10.1016/j.yebeh.2011.08.031 22078515 PMC3713785

[B51] Klimesch W. (1999). EEG alpha and theta oscillations reflect cognitive and memory performance: a review and analysis. Brain Res. Rev. 29, 169–195. 10.1016/S0165-0173(98)00056-3 10209231

[B52] Klimesch W. Doppelmayr M. Russegger H. (1998). Induced alpha band power changes in the human EEG and attention. Neurosci. Lett. 244, 73–76. 10.1016/S0304-3940(98)00122-0 9572588

[B53] Klimesch W. Schimke H. Pfurtscheller G. (1993). Alpha frequency, cognitive load and memory performance. Brain Topogr. 5, 241–251. 10.1007/BF01128991 8507550

[B54] Krupski A. Boyle P. R. (1978). An observational analysis of children's behavior during a simple-reaction-time task: the role of attention. Child Dev. 6, 340–347. 10.2307/1128696

[B55] Li D. Li X. Liang Z. Voss L. J. Sleigh J. W. (2010). Multiscale permutation entropy analysis of EEG recordings during sevoflurane anesthesia. J. Neural Eng. 7, 046010. 10.1088/1741-2560/7/4/046010 20581428

[B56] Li W. Ming D. Xu R. Ding H. Qi H. Wan B. (2012). “Research on visual attention classification based on EEG entropy parameters,” in World Congress on Medical Physics and Biomedical Engineering May 26-31, 2012, Beijing, China. Berlin: Springer, 1553–1556. 10.1007/978-3-642-29305-4_408

[B57] Li Y. Li X. Ratcliffe M. Liu L. Qi Y. Liu Q. (2011). “A real-time EEG-based BCI system for attention recognition in ubiquitous environment,” in Proceedings of 2011 International Workshop on Ubiquitous Affective Awareness and Intelligent Interaction (New York, NY: IOP Publishing), 33–40. 10.1145/2030092.2030099

[B58] Lotte F. Bougrain L. Cichocki A. Clerc M. Congedo M. Rakotomamonjy A.. (2018). A review of classification algorithms for EEG-based brain computer interfaces: a 10 year *Update Univ. S C. Dep. Music*. 15, 031005. 10.1088/1741-2552/aab2f2 29488902

[B59] Luo H. Qiu T. Liu C. Huang P. (2019). Research on fatigue driving detection using forehead EEG based on adaptive multi-scale entropy. Biomed. Signal Process. Control 51, 50–58. 10.1016/j.bspc.2019.02.005

[B60] Lykken D. Tellegen A. Thorkelson K. (1974). Genetic determination of EEG frequency spectra. Biol. Psychol. 1, 245–259. 10.1016/0301-0511(74)90001-5 4473236

[B61] Mardi Z. Ashtiani S. N. M. Mikaili M.. (2011). EEG-based drowsiness detection for safe driving using chaotic features and statistical tests. J. Medical Signals Sens. 1, 130. 10.4103/2228-7477.95297 22606668 PMC3342623

[B62] Márton L. Brassai S. T. Bakó L. Losonczi L. (2014). Detrended fluctuation analysis of EEG signals. Procedia Technol. 12, 125–132. 10.1016/j.protcy.2013.12.46534107041

[B63] Moghaddari M. Lighvan M. Z. Danishvar S. (2020). Diagnose ADHD disorder in children using convolutional neural network based on continuous mental task EEG. Comput. Methods Programs Biomed. 197, 105738. 10.1016/j.cmpb.2020.105738 32927404

[B64] Mohammadpour M. Mozaffari S. (2017). “Classification of EEG-based attention for brain computer interface,” in 2017 3rd Iranian Conference on Intelligent Systems and Signal Processing (ICSPIS) (IEEE), 34–37. 10.1109/ICSPIS.2017.8311585

[B65] Mormann F. Andrzejak R. G. Elger C. E. Lehnertz K. (2007). Seizure prediction: the long and winding road. Brain. 130, 314–333. 10.1093/brain/awl241 17008335

[B66] O'Toole J. M. Temko A. Stevenson N. (2014). “Assessing instantaneous energy in the EEG: a nonnegative, frequencyweighted energy operator,” in 2014 36th Annual International Conference of the IEEE Engineering in Medicine and Biology Society. Chicago, IL: IEEE, 3288–3291. 10.1109/EMBC.2014.6944325 25570693

[B67] Päivinen N. Lammi S. Pitkänen A. Nissinen J. Penttonen M. Grönfors T. (2005). Epileptic seizure detection: a nonlinear viewpoint. Comput. Methods Programs Biomed. 79, 151–159. 10.1016/j.cmpb.2005.04.006 16005102

[B68] Papadelis C. Kourtidou-Papadeli C. Bamidis P. D. Chouvarda I. Koufogiannis D. Bekiaris E.. (2006). “Indicators of sleepiness in an ambulatory EEG study of night driving,” in 2006 International Conference of the IEEE Engineering in Medicine and Biology Society (Shahrood: IEEE), 6201–6204. 10.1109/IEMBS.2006.259614 17946748

[B69] Petersen S. E. Posner M. I. (2012). The attention system of the human brain: 20 years after. Annu. Rev. Neurosci. 35, 73–89. 10.1146/annurev-neuro-062111-150525 22524787 PMC3413263

[B70] Pollock V. Schneider L. Lyness S. (1990). EEG amplitudes in healthy, late-middle-aged and elderly adults: normality of the distributions and correlations with age. Electroencephalogr. Clin. Neurophysiol. 75, 276–288. 10.1016/0013-4694(90)90106-T 1691076

[B71] Prinzel L. J. Pope A. T. Freeman F. G. Scerbo M. W. Mikulka P. J. (2001). “Empirical analysis of EEG and erps for psychophysiological adaptive task allocation,” in NASA Technical Report TM. Available online at: https://ntrs.nasa.gov/citations/20010060403

[B72] Rao R. P. (2013). Brain-Computer Interfacing: an Introduction. Cambridge: Cambridge University Press. 10.1017/CBO9781139032803

[B73] Ray W. J. Cole H. W. (1985). EEG alpha activity reflects attentional demands, and beta activity reflects emotional and cognitive processes. Science. 228, 750–752. 10.1126/science.3992243 3992243

[B74] Roberts S. J. Penny W. Rezek I. (1999). Temporal and spatial complexity measures for electroencephalogram based brain-computer interfacing. Medi. Biol. Eng. Comput. 37, 93–98. 10.1007/BF02513272 10396848

[B75] Robinson J. (2021). “Edinburgh handedness inventory,” in Encyclopedia of Autism Spectrum Disorders. Berlin: Springer, 1600–1604. 10.1007/978-3-319-91280-6_877

[B76] Rosso O. A. Blanco S. Yordanova J. Kolev V. Figliola A. Schürmann M.. (2001). Wavelet entropy: a new tool for analysis of short duration brain electrical signals. J. Neurosci. Methods. 105, 65–75. 10.1016/S0165-0270(00)00356-3 11166367

[B77] Schwender D. Daunderer M. Mulzer S. Klasing S. Finsterer U. Peter K. (1996). Spectral edge frequency of the electroencephalogram to monitor “depth” of anaesthesia with isoflurane or propofol. Br. J. Anaesth. 77, 179–184. 10.1093/bja/77.2.179 8881621

[B78] Solnik S. Rider P. Steinweg K. DeVita P. Hortobgyi T. (2010). Teager kaiser energy operator signal conditioning improves EMG onset detection. Eur. J. Appl. Physiol. 110, 489–498. 10.1007/s00421-010-1521-8 20526612 PMC2945630

[B79] Spasic S. Kesic S. Kalauzi A. Saponjic J. (2011). Different anesthesia in rat induces distinct inter-structure brain dynamic detected by higuchi fractal dimension. Fractals. 19, 113–123. 10.1142/S0218348X1100521X

[B80] Srinivasan V. Eswaran C. Sriraam N. (2007). Approximate entropy-based epileptic EEG detection using artificial neural networks. IEEE Trans. Inf. Technol. Biomed. 11, 288–295. 10.1109/TITB.2006.884369 17521078

[B81] Swartwood J. N. Swartwood M. O. Lubar J. F. Timmermann D. L. (2003). EEG differences in ADHD-combined type during baseline and cognitive tasks. Pediatr. Neurol. 28, 199–204. 10.1016/S0887-8994(02)00514-3 12770673

[B82] Teixeira C. Direito B. Feldwisch-Drentrup H. Valderrama M. Costa R. Alvarado-Rojas C.. (2011). EPILAB: a software package for studies on the prediction of epileptic seizures. J. Neurosci. Methods 200, 257–271. 10.1016/j.jneumeth.2011.07.002 21763347

[B83] Thongpanja S. Phinyomark A. Phukpattaranont P. Limsakul C. (2013). Mean and median frequency of EMG signal to determine muscle force based on time-dependent power spectrum. Elektronika ir Elektrotechnika. 19, 51–56. 10.5755/j01.eee.19.3.3697

[B84] Tong S. Bezerianos A. Malhotra A. Zhu Y. Thakor N. (2003). Parameterized entropy analysis of EEG following hypoxic-ischemic brain injury. Physics Letters A. 314, 354–361. 10.1016/S0375-9601(03)00949-6

[B85] Van Hese P. Philips W. De Koninck J. Van de Walle R. Lemahieu I. (2001). “Automatic detection of sleep stages using the EEG,” in 2001 Conference Proceedings of the 23rd Annual International Conference of the IEEE Engineering in Medicine and Biology Society. Istanbul: IEEE, 1944–1947.24111206

[B86] Vourkas M. Micheloyannis S. Papadourakis G. (2000). “Use of ann and hjorth parameters in mental-task discrimination,” in 2000 First International Conference Advances in Medical Signal and Information Processing (IEE Conf. Publ. No. 476). Bristol: IET, 327–332. 10.1049/cp:20000356

[B87] Wan W. Cui X. Gao Z. Gu Z. (2021). Frontal EEG-based multi-level attention states recognition using dynamical complexity and extreme gradient boosting. Front. Hum. Neurosci. 15, 673955. 10.3389/fnhum.2021.673955 34140885 PMC8204057

[B88] Wang D. Qian M. Fang Y. Lai C. Li D. Chen G. (1989). Revision on the Combined Raven's test for the Rural in China. Psychol. Sci. 5, 004.33148225

[B89] Wang Y. Guan L. (2008). Recognizing human emotional state from audiovisual signals. IEEE Transact. Multimed. 10, 936–946. 10.1109/TMM.2008.927665

[B90] Zheng W.-L. Zhu J.-Y. Lu B.-L. (2017). Identifying stable patterns over time for emotion recognition from EEG. IEEE Transact. Affective Comp. 10, 417–429. 10.1109/TAFFC.2017.271214334292629

[B91] Zoefel B. Huster R. J. Herrmann C. S. (2011). Neurofeedback training of the upper alpha frequency band in EEG improves cognitive performance. Neuroimage. 54, 1427–1431. 10.1016/j.neuroimage.2010.08.078 20850552

